# De novo designed proteins neutralize lethal snake venom toxins

**DOI:** 10.1038/s41586-024-08393-x

**Published:** 2025-01-15

**Authors:** Susana Vázquez Torres, Melisa Benard Valle, Stephen P. Mackessy, Stefanie K. Menzies, Nicholas R. Casewell, Shirin Ahmadi, Nick J. Burlet, Edin Muratspahić, Isaac Sappington, Max D. Overath, Esperanza Rivera-de-Torre, Jann Ledergerber, Andreas H. Laustsen, Kim Boddum, Asim K. Bera, Alex Kang, Evans Brackenbrough, Iara A. Cardoso, Edouard P. Crittenden, Rebecca J. Edge, Justin Decarreau, Robert J. Ragotte, Arvind S. Pillai, Mohamad Abedi, Hannah L. Han, Stacey R. Gerben, Analisa Murray, Rebecca Skotheim, Lynda Stuart, Lance Stewart, Thomas J. A. Fryer, Timothy P. Jenkins, David Baker

**Affiliations:** 1 Department of Biochemistry, University of Washington, Seattle, WA USA; 2 Institute for Protein Design, University of Washington, Seattle, WA USA; 3 Graduate Program in Biological Physics, Structure and Design, University of Washington, Seattle, WA USA; 4 Department of Biotechnology and Biomedicine, Technical University of Denmark, Kongens Lyngby, Denmark; 5 Department of Biological Sciences, University of Northern Colorado, Greeley, CO USA; 6 Centre for Snakebite Research & Interventions, Liverpool School of Tropical Medicine, Pembroke Place, Liverpool, UK; 7 Centre for Drugs & Diagnostics, Liverpool School of Tropical Medicine, Pembroke Place, Liverpool, UK; 8 Biomedical & Life Sciences, Faculty of Health and Medicine, Lancaster University, Lancaster, UK; 9 Sophion Bioscience, Ballerup, Denmark; 10 Department of Infection Biology and Microbiomes, Institute of Infection, Veterinary and Ecological Sciences, University of Liverpool, Liverpool, UK; 11 Media Lab, Massachusetts Institute of Technology, Cambridge, MA USA; 12 Howard Hughes Medical Institute, University of Washington, Seattle, WA USA

**Keywords:** Drug development, Recombinant protein therapy

## Abstract

Snakebite envenoming remains a devastating and neglected tropical disease, claiming over 100,000 lives annually and causing severe complications and long-lasting disabilities for many more^1,2^. Three-finger toxins (3FTx) are highly toxic components of elapid snake venoms that can cause diverse pathologies, including severe tissue damage^3^ and inhibition of nicotinic acetylcholine receptors, resulting in life-threatening neurotoxicity^4^. At present, the only available treatments for snakebites consist of polyclonal antibodies derived from the plasma of immunized animals, which have high cost and limited efficacy against 3FTxs^5–7^. Here we used deep learning methods to de novo design proteins to bind short-chain and long-chain α-neurotoxins and cytotoxins from the 3FTx family. With limited experimental screening, we obtained protein designs with remarkable thermal stability, high binding affinity and near-atomic-level agreement with the computational models. The designed proteins effectively neutralized all three 3FTx subfamilies in vitro and protected mice from a lethal neurotoxin challenge. Such potent, stable and readily manufacturable toxin-neutralizing proteins could provide the basis for safer, cost-effective and widely accessible next-generation antivenom therapeutics. Beyond snakebite, our results highlight how computational design could help democratize therapeutic discovery, particularly in resource-limited settings, by substantially reducing costs and resource requirements for the development of therapies for neglected tropical diseases.

## Abstract (web summary)

Deep learning methods have been used to design proteins that can neutralize the effects of three-finger toxins found in snake venom, which could lead to the development of safer and more accessible antivenom treatments.

## Main

Snakebite envenoming represents a public health threat in many developing regions, notably low-resource settings in sub-Saharan Africa, South Asia, Papua New Guinea and Latin America^2^. With over two million annual cases, snakebites result in 100,000 fatalities and 300,000 permanent disabilities^1^. In 2017, the World Health Organization listed snakebite envenoming as a highest-priority neglected tropical disease^8^. Nonetheless, limited resources have been dedicated to improving the current antivenom treatments^2^. These therapies rely on plasma-derived polyclonal antibodies from hyperimmunized animals, complemented by medical and surgical care^9^. Although instrumental in saving lives, antivenom accessibility is hindered by high production costs and inadequate cold-chain infrastructure in remote areas^9^. Serious adverse effects, including anaphylaxis and pyrogenic reactions, represent additional challenges during antivenom administration^2,10,11^. Furthermore, these treatments are often ineffective in counteracting neurotoxicity and tissue necrosis owing to suboptimal concentrations of neutralizing antibodies against three-finger toxins (3FTxs)^5–7^. This inefficacy stems from the limited immunogenicity of 3FTxs in antivenom-producing animals, resulting in a failure to elicit a strong antibody response^12^. Additional issues arise because of the delayed administration of antivenom treatment^13^. Antibody^14–21^ and non-antibody-based therapeutics^22–28^ have been tested in preclinical studies, but the development of these types of molecules requires either immunization of animals or the development of large libraries that require extensive selection, screening and optimization efforts^29^.

We reasoned that de novo design approaches could have advantages over the traditional methods of antivenom development. First, de novo protein design does not rely on animal immunization and yields proteins that can be manufactured using recombinant DNA technology, thereby creating a source for the continuous production of products with limited batch-to-batch variation. Second, computational design enables the creation of binding proteins with high affinity and specificity without needing extensive experimental screening programmes that often rely on pure toxins, which can be challenging to isolate from whole venoms or generate via recombinant expression^30^. Third, the small size of the designed proteins could offer enhanced tissue penetration^31^ compared to large antibodies, enabling rapid toxin neutralization and thereby being more effective in neutralizing local tissue damage. Fourth, designed proteins can have high thermal stability^32^ and can be produced using low-cost microbial fermentation strategies, which could help enable the development and deployment of new antivenom therapeutics at reduced cost^33^. Hence, we used the deep learning-based RFdiffusion method^32^ to design antivenoms for short-chain and long-chain α-neurotoxins and cytotoxins from the 3FTx snake venom toxin family. We explored the design of binders for both individual natural toxins and consensus toxins representing a family of toxin molecules because binders to the latter could have broader neutralization activity.

## Design of α-neurotoxin-binding proteins

**Fig. 1:**
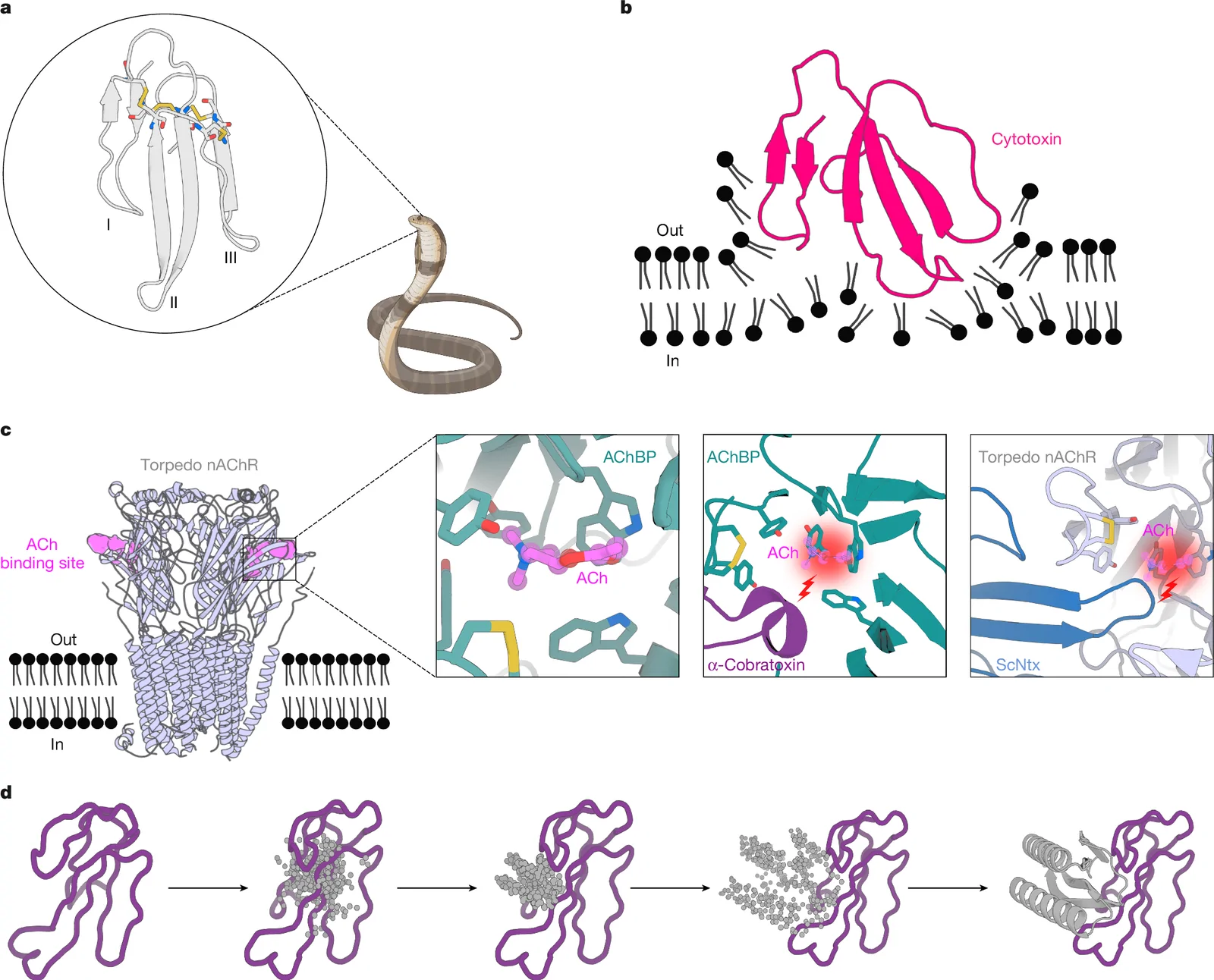
Targets of 3FTxs. **a**, Structure of 3FTxs^59^ (Protein Data Bank (PDB) 1QKD). Highly conserved cysteine residues are highlighted in sticks, and each of the three fingers is indicated (I–III). **b**, Representation of type IA cytotoxin^60^ (dark pink) (PDB 5NQ4) interacting with a lipid bilayer. **c**, Muscle acetylcholine (ACh) (torpedo) receptor (light blue) (PDB 7Z14)^36^. The ACh-binding site is depicted in violet. Left inset: close-up of the acetylcholine-binding protein (AChBP) (teal) (PDB 3WIP) bound to ACh^61^ (violet). A set of aromatic residues form a cage around the neurotransmitter. Middle inset: close-up of α-cobratoxin (dark purple) blocking access to the ACh-binding site in AChBP (teal) (PDB 1YI5)^44^. A molecule of ACh is depicted to illustrate its binding site. Right inset: close-up of ScNtx (dark blue) blocking access to the ACh-binding site in the torpedo receptor (light blue) (PDB 7Z14)^36^. A molecule of ACh is depicted to illustrate its binding site. **d**, Schematic showing α-cobratoxin binder design using RFdiffusion. Starting from a random distribution of residues around the specified β-strands in the target toxin (dark purple), successive RFdiffusion denoising steps progressively removed the noise leading at the end of the trajectory to a folded structure interacting with α-cobratoxin β-strands. Schematics in **a**–**c** were created using BioRender (https://biorender.com).

α-Neurotoxins, a prominent subclass of 3FTxs, adopt a multistranded β-structure with three extended loops protruding from a hydrophobic compact core stabilized by highly conserved disulfide bridges^34,35^ (Fig. 1a). Short-chain and long-chain α-neurotoxins differ in their length and number of disulfide bonds. Despite sequence homology, α-neurotoxins have distinct pharmacological profiles across nicotinic acetylcholine receptor (nAChR) subtypes; short-chain and long-chain α-neurotoxins inhibit muscle-type nAChRs, but only long-chain α-neurotoxins strongly bind to neuronal α7-nAChRs^36^ (Fig. 1c). The venoms of many elapid snake species derive their lethal effects from these toxins, and it is crucial to neutralize both types of α-neurotoxins to achieve therapeutic efficacy and prevent venom-induced lethality.

To achieve functional neutralization, we focused on binding modes that block neurotoxin binding to nAChRs through steric hindrance. Rather than focusing on the nAChR-binding site like previously discovered antibodies^14,20,21^, we took advantage of recently developed methods which enable the robust design of high affinity binders to polar targets by complementing edge β-strands in the target with geometrically matching edge β-strands in the designed binder (ref. ^37^; see Methods). RFdiffusion trajectories were carried out conditioned on secondary structure and block adjacency tensors guiding generation towards each exposed (edge) β-strand in each neurotoxin one at a time. Following generation of backbones making extended β-sheets with the targets through these conditioned RFdiffusion denoising trajectories, sequence design was carried out using ProteinMPNN. The resulting designs were filtered on the basis of AlphaFold2 (AF2) initial guess^38^ and Rosetta metrics, and the most promising candidates were selected for experimental characterization. Top candidates were selected for in vitro validation on the basis of their ability to interfere with toxin binding to nAChR, guided by structural alignments comparing the neurotoxin–nAChR complex with and without the designed binder.

**Fig. 2:**
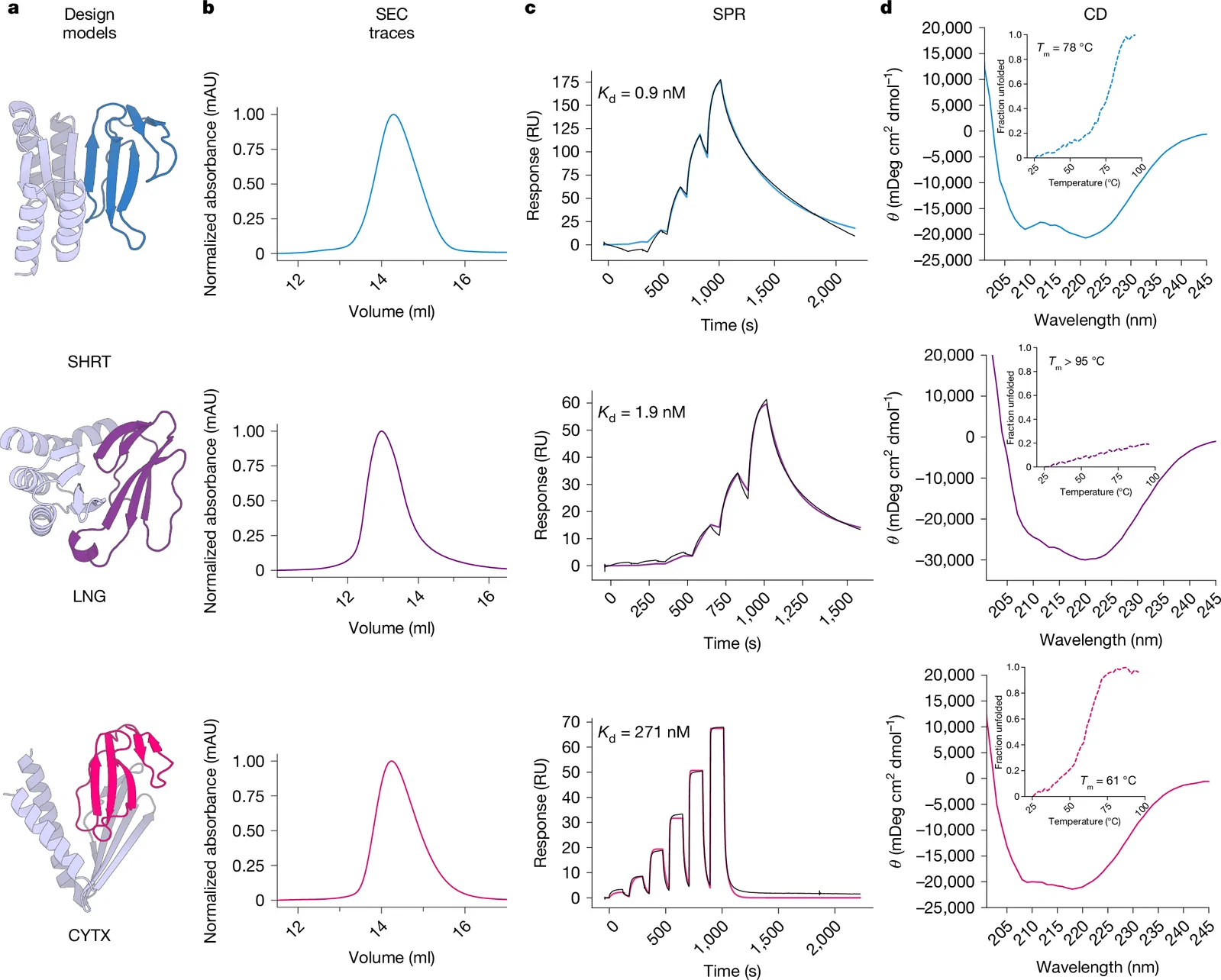
Experimental characterization of 3FTx-binding proteins. **a**, Design models of protein binders (grey) bound to their 3FTx targets (dark blue, ScNtx; dark purple, α-cobratoxin; dark pink, consensus cytotoxin). **b**, SEC traces of purified proteins. mAU, milli-absorbance units. **c**, SPR-binding affinity measurements. Coloured solid lines represent fits using the heterogeneous ligand model, with the dissociation constant (*K*_d_) values derived from these fits. RU, response units. **d**, CD data confirmed the presence of an αβ-secondary structure in the 3FTx-binding proteins and their thermal stability (inset). *θ*, molar ellipticity.

**Fig. 3:**
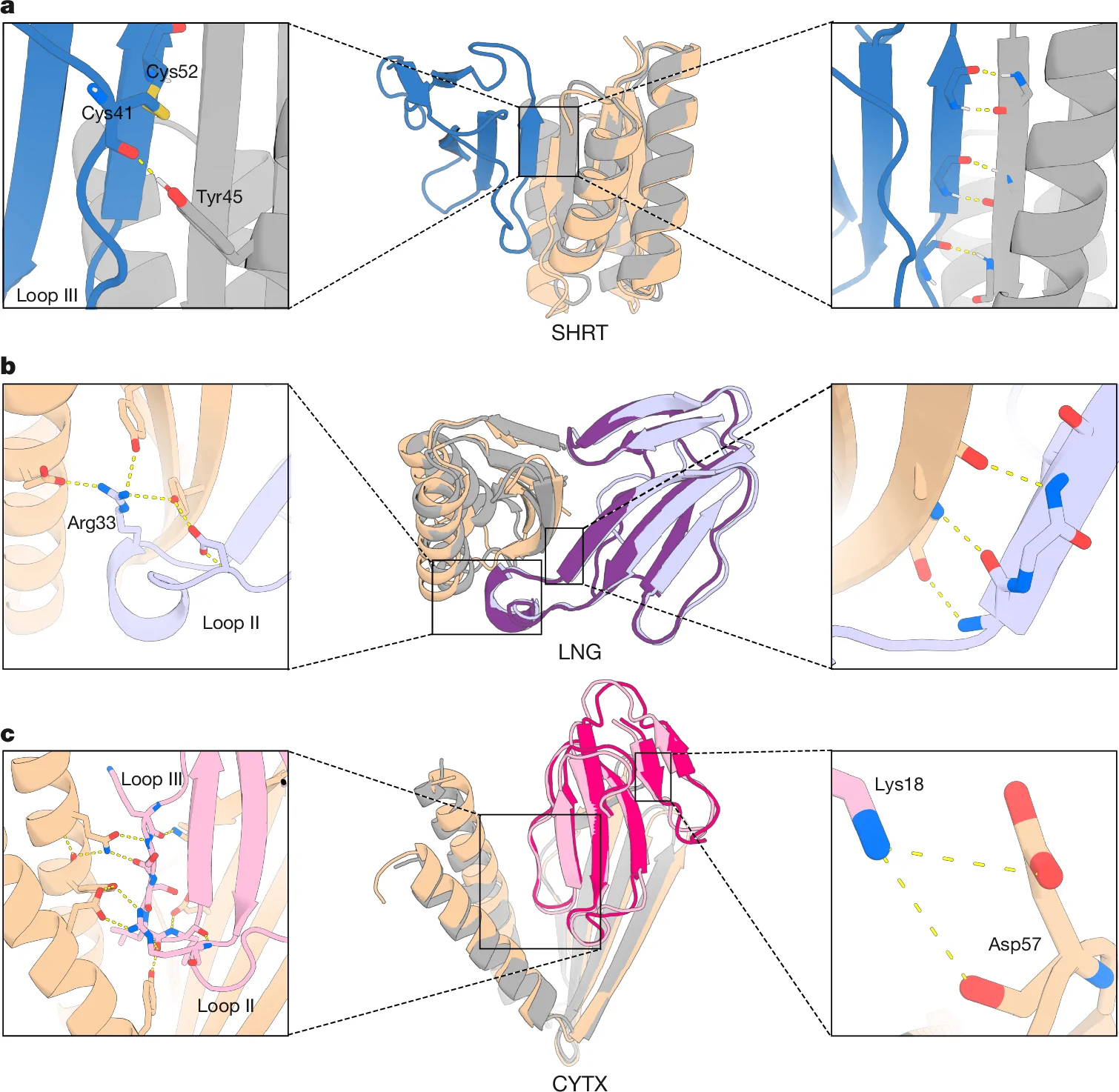
Crystal structures of 3FTx-binding proteins closely matched those of the design models. **a**, Apo-state crystal structure of SHRT design. Left: hydrogen bonding between the carbonyl oxygen of Cys41 in ScNtx (dark blue) and the side chain of Tyr45 in the SHRT design model (grey). Middle: overlay of the SHRT design model (grey) with crystal structure (wheat). Right: backbone hydrogen bonding between the SHRT design model (grey) and ScNtx (dark blue) β-strands. **b**, Crystal structure of LNG design in complex with α-cobratoxin. Left: cross-interface hydrogen-bond network involving Arg33 in loop II of α-cobratoxin (light purple) and Glu69, Tyr40 and Tyr49 in the LNG crystal structure (wheat). Middle: overlay of the LNG design model (grey) bound to α-cobratoxin (dark purple) with crystal structure of binder (wheat) bound to toxin (light purple). Right: backbone hydrogen bonding between the crystal structure of the designed binder (wheat) and α-cobratoxin (light purple) β-strands. **c**, Crystal structure of CYTX_B10 design in complex with *Naja pallida* cytotoxin. Left: cross-interface electrostatic interaction network between loops III and II of *N.* *pallida* cytotoxin (light pink) and the binder crystal structure (wheat). Middle: overlay of the CYTX_B10 design model (grey) bound to the toxin (dark pink) with the crystal structure of the binder (wheat) bound to *N.* *pallida* cytotoxin (light pink). Right: salt bridge between positively charged Lys18 in cytotoxin (light pink) and Asp57 in the binder crystal structure (wheat).

We targeted short-chain α-neurotoxins using a previously designed consensus toxin derived from elapid snakes (ScNtx)^39^ as a representative template (Supplementary Fig. 8). Synthetic genes encoding 44 designs targeting ScNtx were screened via yeast surface display, and one candidate was identified to bind ScNtx with a dissociation constant (*K*_d_) of 842 nM, as confirmed by bio-layer interferometry (BLI) (Supplementary Fig. 1). Following partial diffusion optimization^40^, 11 of 78 designs had higher affinity than the initial hit (Supplementary Fig. 9), with the best (SHRT) having a binding affinity of 0.9 nM, as determined by surface plasmon resonance (SPR) (Fig. 2c, top row; a very similar value of 0.7 nM was obtained by BLI (Supplementary Fig. 2)). SHRT showed a single monomeric peak in size exclusion chromatography (SEC), characteristic αβ-protein circular dichroism (CD) spectra and thermal stability with a melting temperature (*T*_m_) of 78 °C (Fig. 2d, top row). Using X-ray crystallography, we determined the structure of the SHRT design in the apo state, which closely matched the computational design model (2.58 Å resolution; 1.04 Å root-mean-square deviation (RMSD)) (Fig. 3a). The binder interacts with loop III of the neurotoxin, which is key for the binding of the toxin to muscle-type nAChRs^41^ (Fig. 3a). A β-strand in SHRT forms extensive backbone hydrogen bonds with an edge β-strand in the toxin (Fig. 3a, right inset), and tyrosine 45, located on an alpha helix in SHRT, forms a backbone hydrogen bond with cysteine 41 on ScNtx (Fig. 3a, left inset).

As a representative native long-chain α-neurotoxin, we chose α‐cobratoxin (P01391) from *Naja kaouthia*, one of the most extensively characterized toxins in the 3FTx family^42^ (Fig. 1). Of 42 RFdiffusion designs against α-cobratoxin, one candidate had a binding affinity of 1.3 µM using BLI (Supplementary Fig. 3). Partial diffusion optimization of the binding interface generated 38 designs (Supplementary Fig. 10), the highest affinity of which, LNG, had a *K*_d_ of 1.9 nM, as measured by SPR (Fig. 2c, middle row; BLI yielded a value of 6.7 nM (Supplementary Fig. 4)). CD melting experiments showed a very high thermal stability (*T*_m_ > 95 °C; Fig. 2d, middle row).

Using X-ray crystallography, we determined the structure of the LNG α‐cobratoxin binder in complex with the target, which closely matched the computational design model (2.68 Å resolution; 0.42 Å RMSD over design, 0.61 Å over toxin; there was a slight deviation in the positioning of the toxin relative to the binder). As in the design model, the binder interacts with the central loop II of the neurotoxin, which is crucial for the interaction of the toxin with muscle-type and neuronal α7-nAChRs^41,43^. This interaction is primarily mediated by backbone hydrogen bonding between the β-strand in LNG and a β-strand in the toxin (Fig. 3b). Arg33, located at the tip of loop II of α-cobratoxin, forms extensive interactions with LNG (Fig. 3b, left inset). This toxin residue also interacts extensively with AChBP^44^.

## Design of cytotoxin-binding proteins

Cytotoxins, a prominent functional group in the 3FTx family found in cobra venoms, exert cytotoxic effects and induce local tissue damage by destabilizing phospholipid membranes^45^ (Fig. 1b). Neutralizing these toxins is crucial to prevent severe sequelae, such as limb deformity, amputation and lasting disabilities in snakebite victims^46^.

To target cytotoxins, we hypothesized that relying solely on β-strand pairing interactions might not adequately prevent cytotoxin insertion into membranes because of the critical role of their three-finger loops in membrane interaction and disruption^47–50^ (Fig. 1b). Instead, we focused on binding directly to the three-finger loops of the cytotoxin by generating RFdiffusion-based protein backbones with hotspot residues defined in these regions (Fig. 2a, bottom row). To increase the breadth of neutralization, we targeted a consensus sequence derived from 86 different snake cytotoxins (type IA cytotoxin sub-subfamily; Methods). Following ProteinMPNN and AF2 screening, partial diffusion was used to further optimize the designs with the best metrics. A total of 55 protein designs were recombinantly expressed using *Escherichia coli*, and following SEC purification, 18 designs with monomeric populations were tested in a luminescent cell viability assay. Of these, one protein binder (CYTX) had high solubility, with a single monomeric peak in SEC and high neutralization activity against *Naja pallida* and *Naja nigricollis* whole venoms, known for their high cytotoxin content^51^ (Supplementary Fig. 5). The *K*_d_ for the cytotoxin from *N.* *pallida* was determined to be 271 nM via SPR (Fig. 2c, bottom row). CYTX exhibited a characteristic αβ-protein CD spectrum and was thermostable with a *T*_m_ of 61 °C (Fig. 2d, bottom row).

Few designed binders have targeted loops; hence, we sought to solve the crystal structure of CYTX in complex with the *N.* *pallida* cytotoxin (Fig. 3c). To reduce flexibility to favour crystallization, a disulfide bond was introduced in a flexible loop connecting the β-sheet segment to the two α-helices of CYTX, yielding a candidate (CYTX_B10) with improved thermal stability (*T*_m_ = 70.3 °C) and monomeric profile during SEC, but a slightly weaker *K*_d_ of 740 nM for *N.* *pallida* cytotoxin (Supplementary Fig. 6). The structure of CYTX_B10 in complex with the target closely matched the computational design model (resolution, 2.0 Å; RMSD, 1.32 Å over design and 0.58 Å over toxin), showing extensive electrostatic interactions involving side chain–main chain hydrogen bonds between cytotoxin loops II and III and the CYTX_B10 binder (Fig. 3c, left inset). The unusual open fold of CYTX_B10 highlights the power of RFdiffusion to custom generate scaffolds shape-matched with protein targets and the power of proteinMPNN to stabilize structures that violate common rules of protein structure (in this case, lacking a central hydrophobic core).

## In vitro neutralization

**Fig. 4:**
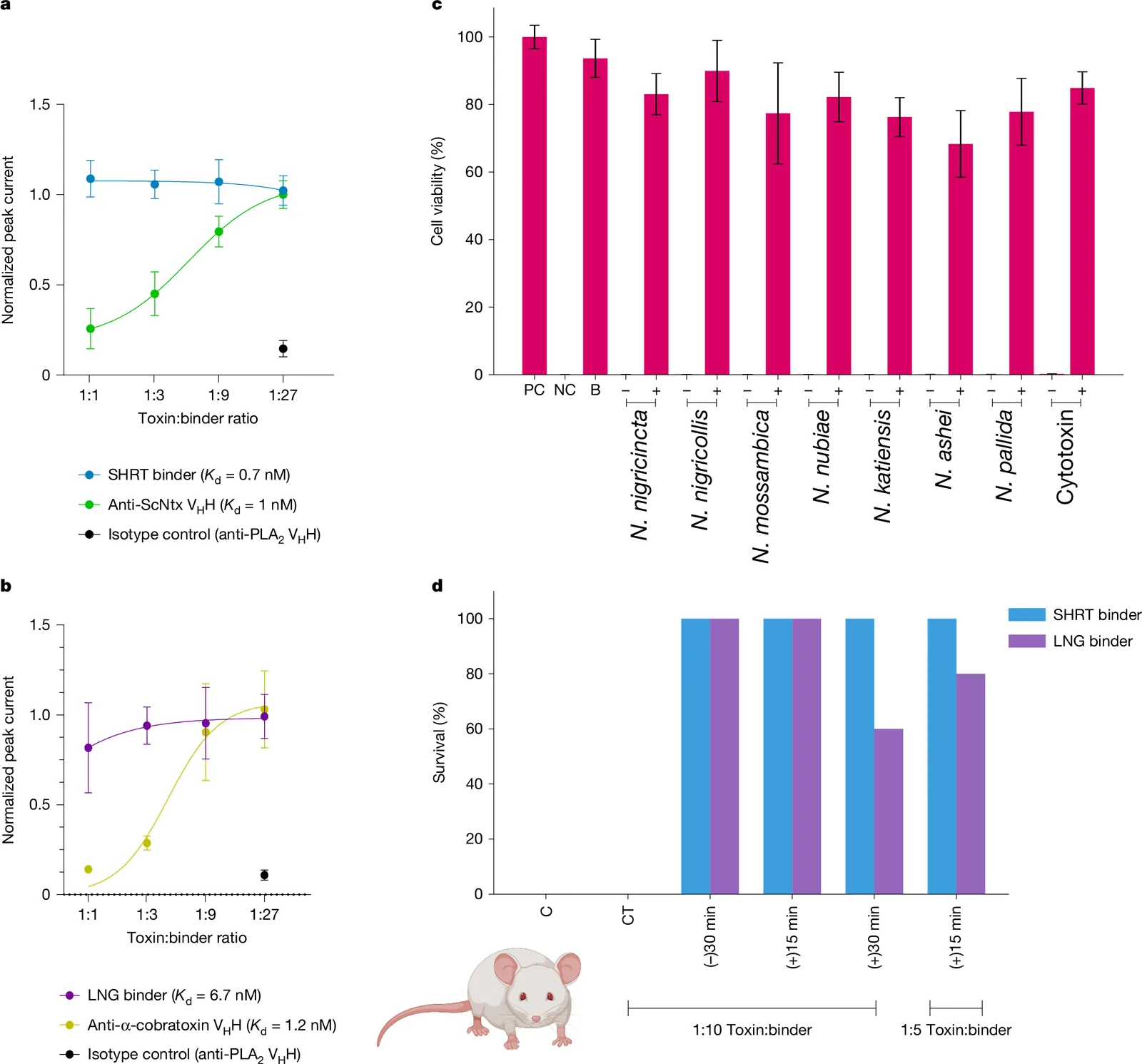
In vitro and in vivo efficacy. **a**, Concentration–response curves comparing SHRT binder and an anti-ScNtx nanobody (V_H_H) efficacy in preventing nAChR blocking by ScNtx at a dose corresponding to the 80% inhibitory concentration (IC_80_). The *y* axis corresponds to the ACh response in the presence of toxin and design, normalized to full ACh response, and averaged in each group (*n* = 16). **b**, Concentration–response curves comparing the efficacy of the LNG binder and anti-α-cobratoxin V_H_H in preventing nAChR blocking by one IC_80_ of α-cobratoxin. **c**, Neutralization of the cytolytic effects of whole venoms from seven different *Naja* species and isolated cytotoxin by the CYTX binder. Two IC_50_ values of the whole venom or toxin were preincubated with CYTX at a 1:5 molar ratio (toxin:binder). This ratio was estimated assuming that 70% of the whole venom consists of cytotoxins, on the basis of previous proteomic analyses^53^. Keratinocyte medium was used as a positive control (PC). Triton X-100 was used as a negative control (NC). CYTX binder (B) was used as a PC. (−) denotes two IC_50_ values of the whole venom without a binder, and (+) denotes venom incubated with a binder. Experiments were performed in triplicate, and the results are expressed as mean ± s.d. **d**, Survival of mice following lethal neurotoxin challenge (*n* = 5). At a concentration corresponding to three times the LD_50_ value of ScNtx or α-cobratoxin, either were preincubated for 30 min (−30 min) with the corresponding protein binders at a 1:10 ratio and then administered intraperitoneally into groups of five mice. Toxins were administered intraperitoneally following IP administration of binders at 1:10 or 1:5 molar ratios (toxin:binder) either 15 min (+15 min) or 30 min (+30 min) post-toxin injection. Controls included mice that received the toxins alone (C). Specificity was assessed via cross-treatment (CT) experiments, in which non-target binders were preincubated with three LD_50_ values of ScNtx or α-cobratoxin and administered intraperitoneally. Signs of toxicity were observed, and deaths were recorded for a period of 24 h. Illustration in **d** created with BioRender (https://biorender.com).

We assessed the ability of the designs to functionally neutralize α-neurotoxins in patch-clamp experiments using a human-derived rhabdomyosarcoma cell line expressing muscle-type nAChRs. When preincubated with ScNtx, the SHRT design achieved complete neutralization at a 1:1 molar ratio (toxin:binder), which was better than the previously characterized ScNtx nanobody (TPL1163_02_A01)^52^ (Fig. 4a; a control nanobody targeting phospholipase A_2_ had no effect). Similarly, the LNG design had a better neutralizing efficacy than a previously characterized α-cobratoxin nanobody (TPL1158_01_C09)^52^, achieving full protection at a 1:1 molar ratio (toxin:binder) (Fig. 4b).

We used a cytotoxicity assay to evaluate the cross-reactivity of the CYTX design against various cobra whole venoms. Immortalized human keratinocytes (N/TERTs) were exposed to venoms from seven different *Naja* species, which previous proteomic analyses suggested primarily consist (approximately 70%) of cytotoxins^53^. Preincubating CYTX with venom (two IC_50_ values) at a 1:5 molar ratio (toxin:binder) provided 70–90% protection against venom-induced cytotoxicity (Fig. 4c). Similarly, preincubation of the cytotoxin binder with the isolated cytotoxin from *N.* *pallida* (two IC_50_ values) at a 1:5 molar ratio (toxin:binder) provided 85% protection against cytotoxicity (Fig. 4c). However, preliminary studies indicated that the CYTX design, in 1:1, 1:2.5 and 1:5 molar ratios (toxin:binder), did not significantly decrease the size of the dermonecrotic lesions induced by intradermal *N.* *nigricollis* venom administration in a murine model^54^ (Supplementary Fig. 7). The affinity of CYTX likely needs to be further optimized for full in vivo neutralization of cytotoxins.

## In vivo protection

Given the encouraging in vitro neutralization of our anti-neurotoxin designs, we proceeded to in vivo studies. We determined the mean lethal dose (LD_50_) values for α‐neurotoxins in male non-Swiss albino mice via intraperitoneal administration; α-cobratoxin had an LD_50_ of 0.098 µg g^−1^ and ScNtx had an LD_50_ of 0.087 µg g^−1^, in agreement with previous intravenous LD_50_ doses of these toxins (0.1 µg g^−1^) (ref. ^55^). To evaluate the in vivo neutralization efficacy of our neurotoxin-targeting designs, we monitored survival for 24 h post-lethal neurotoxin challenge following the administration of purified toxins (three LD_50_ values) (Fig. 4d). The SHRT binder provided complete protection (100%) to mice when preincubated and administered intraperitoneally with the corresponding short-chain neurotoxin at a 1:10 molar ratio of toxin to binder; however, as expected, it did not neutralize the non-target α‐cobratoxin. The LNG binder exhibited comparable efficacy, completely neutralizing α‐cobratoxin but not the non-target ScNtx (Fig. 4d, left). In rescue assays that better mimicked a real-life snakebite scenario, complete protection (100%) was achieved when short-chain or long-chain α-neurotoxin binders were administered intraperitoneally at a 1:10 molar ratio (toxin:binder) 15 min after a lethal α-neurotoxin challenge (three LD_50_ values) (Fig. 4d, middle). Administering the SHRT binder 30 min after toxin injection also provided 100% protection against ScNtx, whereas the LNG binder conferred 60% protection against α‐cobratoxin (Fig. 4d, right). All of the surviving mice showed no evidence of limb or respiratory paralysis. At a 1:5 molar ratio (toxin:binder), intraperitoneal administration of the SHRT design 15 min after toxin injection (three LD_50_ values) resulted in 100% survival, whereas the LNG binder provided 80% protection. Mice injected with the binder alone showed no negative effects at 24 and 48 h post-injection or up to 2 weeks post-injection.

## Discussion

Antivenoms based on animal-derived polyclonal antibodies have long been the cornerstone of snakebite therapy, but their application is hampered by limited efficacy against toxins with low immunogenicity, propensity to cause severe adverse reactions, inherent batch-to-batch variations and high production costs associated with their manufacture^56^. Thus, there has been a search for alternatives, with recombinant human monoclonal antibodies and nanobodies presenting solutions that can help overcome some of these limitations^57^. Our designed neurotoxin binders demonstrate comparable potency to the best immunoglobulin G antibodies and nanobodies reported in the literature^57^ and are highly stable and readily producible in microbial systems. Their small size (approximately 100 amino acids) may enable them to penetrate rapidly into deep tissue^31^. More generally, our in silico design approach avoids animal immunization and/or construction and several rounds of selection and/or screening of large libraries, providing a low-cost methodology for the rapid development of toxin binders to the many components of snake venom when structural or sequence data exist for these targets. De novo designed proteins have high stability and are amenable to low-cost manufacturing, which is key to effectively address snakebite envenoming as a neglected tropical disease. From the design perspective, the crystal structure of our cytotoxin binder highlights the ability of RFdiffusion to custom design scaffolds to match almost any target shape and to generate binders to loop regions of proteins. The inhibitory activity of our anti-cytotoxin designs directly supports a hypothesized role for the cytotoxin loops in membrane disruption.

Advancing the field to provide effective solutions for snakebite victims requires a collaborative effort involving the scientific community, pharmaceutical industry, public health systems, and governments^2^. Although traditional antivenoms will likely remain a therapeutic cornerstone in snakebite treatment for the immediate future, our de novo designed binders could potentially be used as fortifying agents to improve the efficacy of antivenoms, which would be particularly beneficial in the treatment of elapid envenomings, in which low-molecular-mass toxins with limited immunogenicity but high medical importance dominate toxicity and therefore must be neutralized^58^. Beyond fortification, RFdiffusion could be used to generate designs that neutralize other medically relevant toxins, expediting the formulation of antivenoms with broader species coverage. More generally, as in silico protein design is less resource-intensive than traditional antibody development, our approach could aid in the democratization of drug design and discovery, enabling researchers in low-income and middle-income countries to better contribute to the development of effective treatments for snakebite envenoming and other neglected tropical diseases.

## Methods

### Cytotoxin consensus sequence design

Amino acid sequences for cytotoxins were collected from the UniProt website using family: “snake three-finger toxin family Short-chain subfamily Type IA cytotoxin sub-subfamily” as a query. The resultant 86 unique CTX sequences were subjected to multiple sequence alignments in Clustal Omega^62^. Using these alignments, a consensus sequence was designed to represent the most common amino acid at each position across the aligned sequences. In this process, each column of the sequence alignment was analysed to select the most frequent amino acid. In scenarios in which no single amino acid was dominant, a consensus symbol was used to represent a group of similar amino acids on the basis of their properties, such as charge or hydrophobicity. This approach allowed the representation of conserved biochemical properties rather than specific amino acid identities at positions with high variability.

### Secondary structure and block adjacency tensors

To generate the desired binder–target β-strand pairing interactions using RFdiffusion, fold-conditioning tensors describing single binder β-strands interacting with the target β-strands in a matrix format were supplied to RFdiffusion at inference. This information was supplied via two tensors: an [*L*,4] secondary one-hot tensor (0 = α-helix, 1 = β-strand, 2 = loop and 3 = masked secondary structure identity) to indicate the secondary structure classification of each residue in the binder–target complex, and an [*L*,*L*,3] adjacency one-hot tensor (0 = non-adjacent, 1 = adjacent and 2 = masked adjacency) to indicate interacting partner residues for each residue in the binder–target complex. For the design of the binders described here, the secondary structure tensor indicated an entirely masked binder structure, with the exception of binder residues set to β-strand identities, whereas the adjacency tensor indicated a masked adjacency between binder–target residues, with the exception of the predefined strand residues being adjacent to the defined target strand residues.

### De novo 3FTx binder design using RFdiffusion

The crystal structures of ScNtx (PDB 7Z14) and α-cobratoxin (PDB 1YI5) served as the inputs for RFdiffusion. In the case of the consensus cytotoxin, the AF2 model was used. Approximately 2,000 diffused designs were generated for each target, using the secondary structure and block adjacency tensors in the RFdiffusion model. The resulting backbone libraries underwent sequence design using ProteinMPNN, followed by FastRelax and AF2 + initial guess^38^. The resulting libraries were filtered on the basis of AF2 predicted aligned error (PAE) < 10, predicted local distance difference test (pLDDT) > 80 and Rosetta Delta Delta G (ddg) < −40.

### Partial diffusion to optimize binders

The AF2 models of the highest-affinity designs for each toxin target were used as the inputs for partial diffusion. The models were subjected to 10 and 20 noising time steps out of a total of 50 time steps in the noising schedule and subsequently denoised (“diffuser.partial_T” input values of 10 and 20). Approximately 2,000 partially diffused designs were generated for each target. The resulting library of backbones was sequence designed using ProteinMPNN after Rosetta FastRelax, followed by AF2 + initial guess^38^. The resulting libraries were filtered on the basis of AF2 PAE < 10, pLDDT > 80 and Rosetta ddg < −40.

### Recombinant expression of ScNtx

ScNtx was recombinantly expressed from the methylotrophic yeast *Komagataella phaffii* (formerly known as *Pichia pastoris*). The ScNtx sequence was codon-optimized for expression in yeast and included an N-terminal His_6_ tag, followed by a biotin acceptor peptide and a tobacco etch virus proteolytic site. The expression was performed as described previously^30^. The culture medium was dialysed overnight against wash buffer (50 mM sodium phosphate buffer (pH 8.0) and 20 mM imidazole). Purification was carried out using an NGC chromatography system (Bio-Rad) with a 5 ml immobilized metal affinity chromatography (IMAC) nickel column (Bio-Rad). After loading, the column was washed with 5 column volumes of wash buffer to remove non-specifically bound proteins. The protein was then eluted using a gradient of 250 mM imidazole over 10 column volumes. Fractions with a high absorbance at 280 nm were pooled and dialysed against 50 mM sodium phosphate buffer (pH 8.0). Purity was assessed on SDS–PAGE to confirm the size. The protein solution was aliquoted and stored at −20 °C for further use.

### Toxins

α-Cobratoxin (L8114) was obtained from Latoxan. Cytotoxin from *N.* *pallida* was obtained from Sigma-Aldrich (217503).

### Venoms

Whole venoms for initial neutralization screening from *N.* *nigricollis* (CV01089563VEN) and *N.* *pallida* (CV01089566VEN) were obtained in lyophilized form from Amerigo Scientific. Catalogue numbers are provided in parentheses.

For in vitro neutralization experiments in human keratinocytes, whole venoms from *N.* *nigricollis* (L1327), *Naja nigricincta* (L1368), *Naja mossambica* (L1376), *Naja nubiae* (L1342), *Naja katiensis* (L1317), *Naja ashei* (L1375) and *N.* *pallida* (L1321) were purchased in lyophilized form from Latoxan. Catalogue numbers are provided in parentheses.

For the in vivo anti-cytotoxin study, *N.* *nigricollis* venom was sourced from wild-caught Tanzanian specimens housed in the herpetarium of Liverpool School of Tropical Medicine.

### Gene construction of 3FTx binders

The designed protein sequences were optimized for expression in *E.* *coli*. Linear DNA fragments (eBlocks; Integrated DNA Technologies) encoding the design sequences contained overhangs suitable to cloning into the pETcon3 vector for yeast display (Addgene #45121) and LM627 vector for protein expression (Addgene #191551) through Golden Gate cloning.

### Yeast display screening

For yeast transformation, 50–60 ng of pETcon3, digested with NdeI and XhoI restriction enzymes, and 100 ng of the insert (eBlocks) were transformed into *Saccharomyces cerevisiae* EBY100 following the protocol described in a previous study^63^. EBY100 cultures were cultivated in C-Trp-Ura medium with 2% (w/v) glucose. To induce expression, yeast cells initially grown in the C-Trp-Ura medium with 2% (w/v) glucose were transferred to SGCAA medium containing 0.2% (w/v) glucose and induced at 30 °C for 16–24 h. After induction, the cells were washed with PBSF (phosphate-buffered saline (PBS) with 1% (w/v) bovine serum albumin) and labelled for 40 min with biotinylated toxin targets at room temperature using the without-avidity labelling condition^63^. Subsequently, the cells were washed, resuspended in PBSF and individually sorted on the basis of each unique design using a 96-well compatible autosampler in the Attune NxT Flow Cytometer (Thermo Fisher Scientific).

### Protein expression and purification in *E.coli* for 3FTx binders

Protein expression was conducted in 50 ml of Studier autoinduction medium supplemented with kanamycin, and cultures were grown overnight at 37 °C. Cells were collected by centrifugation at 4,000×*g* for 10 min and resuspended in lysis buffer (100 mM Tris-HCl, 200 mM NaCl and 50 mM imidazole) supplemented with Pierce Protease Inhibitor Tablets (EDTA-free). Cell lysis was achieved by sonication using a Qsonica Q500 instrument with a four-pronged horn for 2.5 min ON total at an amplitude of 80%. Soluble fractions were clarified by centrifugation at 14,000×*g* for 40 min and subsequently purified by affinity chromatography using Ni-NTA resin (Qiagen) on a vacuum manifold. Washes were performed using low-salt buffer (20 mM Tris-HCl, 200 mM NaCl and 50 mM imidazole) and high-salt buffer (20 mM Tris-HCl, 1,000 mM NaCl and 50 mM imidazole) before elution with elution buffer (20 mM Tris-HCl, 200 mM NaCl and 500 mM imidazole). Eluted protein samples were filtered and injected into an autosampler-equipped ÄKTA pure system on a Superdex S75 Increase 10/300 GL column at room temperature using SEC running buffer (20 mM Tris-HCl and 100 mM NaCl (pH 8)). Monodisperse peak fractions were pooled, concentrated using spin filters (3 kDa molecular weight cutoff; Amicon; Millipore Sigma) and stored at 4 °C before downstream characterizations. Protein concentrations were determined by measuring absorbance at 280 nm using a NanoDrop spectrophotometer (Thermo Fisher Scientific) using the molecular weights and extinction coefficients obtained from their amino acid sequences using the ProtParam tool.

### BLI binding experiments

BLI experiments were performed on an Octet RED96 (ForteBio) instrument, with streptavidin-coated tips (Sartorius item no. 18-5019). Buffer comprised 1× HBS-EP+ buffer (Cytiva BR100669) supplemented with 0.1% w/v bovine serum albumin. The tips were preincubated in buffer for at least 10 min before use. The tips were then sequentially incubated in biotinylated toxin target, buffer, designed binder and buffer.

### Affinity measurements by SPR

SPR experiments were conducted using a Biacore 8K instrument (Cytiva) and analysed using the accompanying evaluation software. Biotinylated α-cobratoxin was immobilized on a streptavidin sensor chip (Cytiva). For ScNtx and *N.* *pallida* cytotoxin, immobilization involved the activation of carboxymethyl groups on a dextran-coated chip through reaction with N-hydroxysuccinimide. The ligands were then covalently bonded to the chip surface by means of amide linkages, and the excess activated carboxyls were blocked with ethanolamine (https://doi.org/10.1007/978-1-59745-523-7_20). Increasing concentrations of protein binders were flown over the chip in 1× HBS-EP+ buffer (Cytiva BR100669).

### Circular dichroism

The secondary structure content was evaluated by CD in a Jasco J-1500 CD spectrometer coupled to a Peltier system (EXOS) for temperature control. The experiments were performed on quartz cells with an optical path of 0.1 cm, covering a wavelength range of 200–260 nm. The CD signal was reported as molar ellipticity (*θ*). The thermal unfolding experiments were followed by a change in the ellipticity signal at 222 nm as a function of temperature. Proteins were denatured by heating at 1 °C min^−1^ from 20 to 95 °C.

### Crystallization and structure determination

Crystallization experiments for the binder complex were conducted using the sitting drop vapour diffusion method. Crystallization trials were set up in 200 nl drops using a 96-well format by mosquito LCP from SPT Labtech. Crystal drops were imaged using the UVEX crystal plate hotel system by JANSi. Diffraction quality crystals for the LNG binder complex appeared in 1.5 M ammonium sulfate and 25% (v/v) glycerol in 2 weeks. Diffraction quality crystals for the SHRT binder appeared in 0.08 M sodium acetate trihydrate (pH 4.6), 1.6 M ammonium sulfate and 20% (v/v) glycerol. For CYTX_B10-complex diffraction, quality crystals appeared in 0.1 M 2-(*N*-morpholino)ethanesulfonic acid (MES) (pH 6), 0.01 M zinc chloride, 20% (w/v) polyethylene glycol (PEG) 6000 and 10% (v/v) ethylene glycol. The crystals were flash-cooled in liquid nitrogen before being transported to the synchrotron for diffraction experiments.

The diffraction data were collected at the National Synchrotron Light Source II Beamline AMX (17-ID-1). X-ray intensities and data reduction were evaluated and integrated using XDS^64^ and merged/scaled using Pointless/Aimless in the CCP4i2 Program Suite^65^. The structure was determined by molecular replacement using a model designed using Phaser^66^. Following molecular replacement, the model was improved and refined using Phenix^67^. Model building was performed using Coot^68^ in between refinement cycles. The final model was evaluated using MolProbity^69^. Data collection and refinement statistics are reported in Extended Data Table 1. The final atomic coordinates, mmCIF and structural factors were deposited in the PDB with accession codes 9BK5, 9BK6 and 9BK7, respectively.

### In vitro neutralization using electrophysiology

Human-derived rhabdomyosarcoma RD cells (American Type Culture Collection) endogenously expressing the muscle-type nAChR were used for electrophysiology experiments^20^. Planar whole-cell patch-clamp recordings were conducted on a Qube automated electrophysiology platform (Sophion Bioscience) with 384-channel patch chips (patch hole resistance 2.00 ± 0.02 MΩ), following the protocol detailed in a previous study^20^. Protein binders were preincubated with approximately one 80% inhibitory concentration (IC_80_) of α-cobratoxin or ScNtx at various toxin-to-binder molar ratios (1:1, 1:3, 1:9 and 1:27) and then added to the cells. The ability of the toxin to inhibit an acetylcholine (ACh; 70 µM) response in the presence or absence of binders was normalized to the full ACh response, averaged in each group (*n* = 16) and represented in a non-cumulative concentration–response plot. We analysed data using Sophion Analyzer v.6.6.70 (Sophion Bioscience) and GraphPad Prism v.10.1.1 (GraphPad Software).

### Initial neutralization screening of whole venoms using cell viability assay

HEK293T cells were cultured in Dulbecco’s modified Eagle’s medium (Gibco) supplemented with 10% fetal bovine serum at 37 °C and 5% CO_2_. Cells were subjected to commercial whole venoms from *N.* *pallida* (34 µg ml^−1^) and *N.* *nigricollis* (42 µg ml^−1^), either in the absence or presence of 1:1 or 5:1 molar ratio of toxin:binder. Buffer and binder-only controls were run in parallel, and all samples were preincubated for 30 min at room temperature before addition to HEK293T cells. To determine the percentage of viable cells, RealTime-Glo MT Cell Viability Assay (Promega) was performed according to the manufacturer’s protocol. Experiments were performed in triplicate, and the results were expressed as mean ± s.d.

### In vitro neutralization of whole venoms using cell viability assay

N/TERT-immortalized keratinocytes were cultured as described previously^70^. After determining the IC_50_ for seven venoms of *Afronaja* snakes, N/TERT cells were subjected to twice the IC_50_ of each venom, either in the absence or presence of a 1:5 molar ratio of venom:binder. Buffer and binder-only controls were run in parallel, and all samples were preincubated (30 min at 37 °C) before addition to N/TERT cells. To determine the percentage of viable cells, the CellTiter-Glo Luminescent Cell Viability Assay (Promega) was performed according to the manufacturer’s protocol. Experiments were performed in triplicates, and results were expressed as mean ± s.d.

### LD_50_ determinations for α-neurotoxins

All assays used male non-Swiss albino mice (20–30 g), and all doses were mass adjusted. The toxins assayed were α-cobratoxin (7,820 Da, from *N.* *kaouthia* venom obtained from Latoxan S.A.S.) and the short-chain neurotoxin ScNtx (8,944 Da, recombinantly expressed). The toxins were solubilized in PBS at 1.0 mg ml^−1^ and then diluted in PBS as needed. For toxin LD_50_ determination, five doses with three mice per dose were used, and a 100 µl bolus was injected intraperitoneally in the right lower abdominal region; controls received only PBS. The injected mice were observed for the first 2 h and then again at 24 h. LD_50_ values were calculated using the Quest Graph LD_50_ Calculator^71^.

### In vivo neurotoxicity protein binder protection assays

In the preincubation experiments, three LD_50_ values of the toxins (α-cobratoxin, 0.294 µg g^−1^ mouse; ScNtx, 0.261 µg g^−1^ mouse) were mixed with a ten-fold molar excess of their respective protein binders in PBS and incubated at room temperature for 30 min before intraperitoneal administration. Groups of five mice were injected with the binder:toxin mixture and observed at 2 and 24 h. In rescue experiments, toxins (three LD_50_ values) were administered intraperitoneally 15 or 30 min before the corresponding binder, given intraperitoneally at either ten-fold or five-fold molar excess to groups of five mice. Protection against lethality was measured as per cent mortality at 24 h.

### In vivo dermonecrosis protein binder protection assays

CD1 male mice (18–20 g; Charles River Laboratories) were acclimated for 1 week before experimentation in specific pathogen-free conditions. The holding room conditions were 23 °C with 45–65% humidity and 12/12 h light cycles (350 lux). Mice were housed in Tecniplast GM500 cages (floor area of 501 cm^2^) containing 120 g LIGNOCEL wood fibre bedding (JRS) and Z-nest biodegradable paper-based material for nesting and environmental enrichment (red house, clear polycarbonate tunnel and loft). The mice had ad libitum access to irradiated PicoLab food (LabDiet) and reverse osmosis water in an automatic water system. The animals were split into cages (experimental units) upon arrival, and no further randomization was performed.

All mice were pretreated with 5 mg kg^−1^ morphine (injected subcutaneously) before receiving intradermal injections in a 100 μl volume into the ventral abdominal region (rear side flank region). A venom-only control group of five mice received 63 μg of *N.* *nigricollis* (Tanzania) venom (dissolved in PBS). For protection assays, crude venom was preincubated (30 min at 37 °C) with varying cytotoxin:binder ratios of 1:1, 1:2.5 and 1:5 before injection (*n* = 3) (ratios estimated from the proportion of cytotoxin in the venom). Before this, the control group (*N* = 3) received injections of cytotoxin binder alone (278 µM, equivalent to the 1:5 cytotoxin:binder dose) to check tolerance of the cytotoxin binder. For sample size, *N* = 3 was used for groups receiving the cytotoxin binder because this was a pilot experiment. *N* = 5 was used for the venom-only control group because of the variation in lesion size, which is the size recommended by the World Health Organization. In total, 17 mice were used. No inclusion or exclusion criteria were used during the experiment, and all data points were used in the analysis. No strategy was used to control confounders. All experimenters were aware of the group allocation during the experiment and analysis.

After 72 h, the mice were euthanized with increasing concentrations of CO_2_, and the lesions were excised. The outcome measured was the lesion size. Photographs of the lesions were taken using a digital camera immediately after excision, and the severity and size of the dermonecrotic lesions were determined using Venom Induced Dermonecrosis Analysis tooL (VIDAL)^72^.

### Ethical approval

Animal experiments for in vivo neurotoxicity assays were conducted at the University of Northern Colorado under protocol 2303D-SM-S-26, approved by the University of Northern Colorado Institutional Animal Care and Use Committee (UNC-IACUC), in accordance with Government Principles, Public Health Policy, US Department of Agriculture Animal Welfare Act and the Guide for the Care and Use of Laboratory Animals.

Animal experiments for in vivo dermonecrosis assays were approved by the Animal Welfare and Ethics Review Board of the Liverpool School of Tropical Medicine and the University of Liverpool, and conducted under the UK Home Office project licence P58464F90 in accordance with the UK Animal (Scientific Procedures) Act 1986.

### Cell line development, acquisition and authentication

HEK293T cells (American Type Culture Collection CRL-3216) and N/TERT-immortalized keratinocytes, provided by E. O’Toole (Queen Mary University of London), were authenticated by means of morphological assessment and tested for mycoplasma contamination.

### Reporting summary

Further information on research design is available in the Nature Portfolio Reporting Summary linked to this article.

## Online content

Any methods, additional references, Nature Portfolio reporting summaries, source data, extended data, supplementary information, acknowledgements, peer review information; details of author contributions and competing interests; and statements of data and code availability are available at https://doi.org/10.1038/s41586-024-08393-x.

## Supplementary information

Supplementary Information This file contains Supplementary Figs. 1–10, Table 1 and References.

Reporting Summary

Peer Review File

## Figures and Tables

**Extended Data Table 1:**
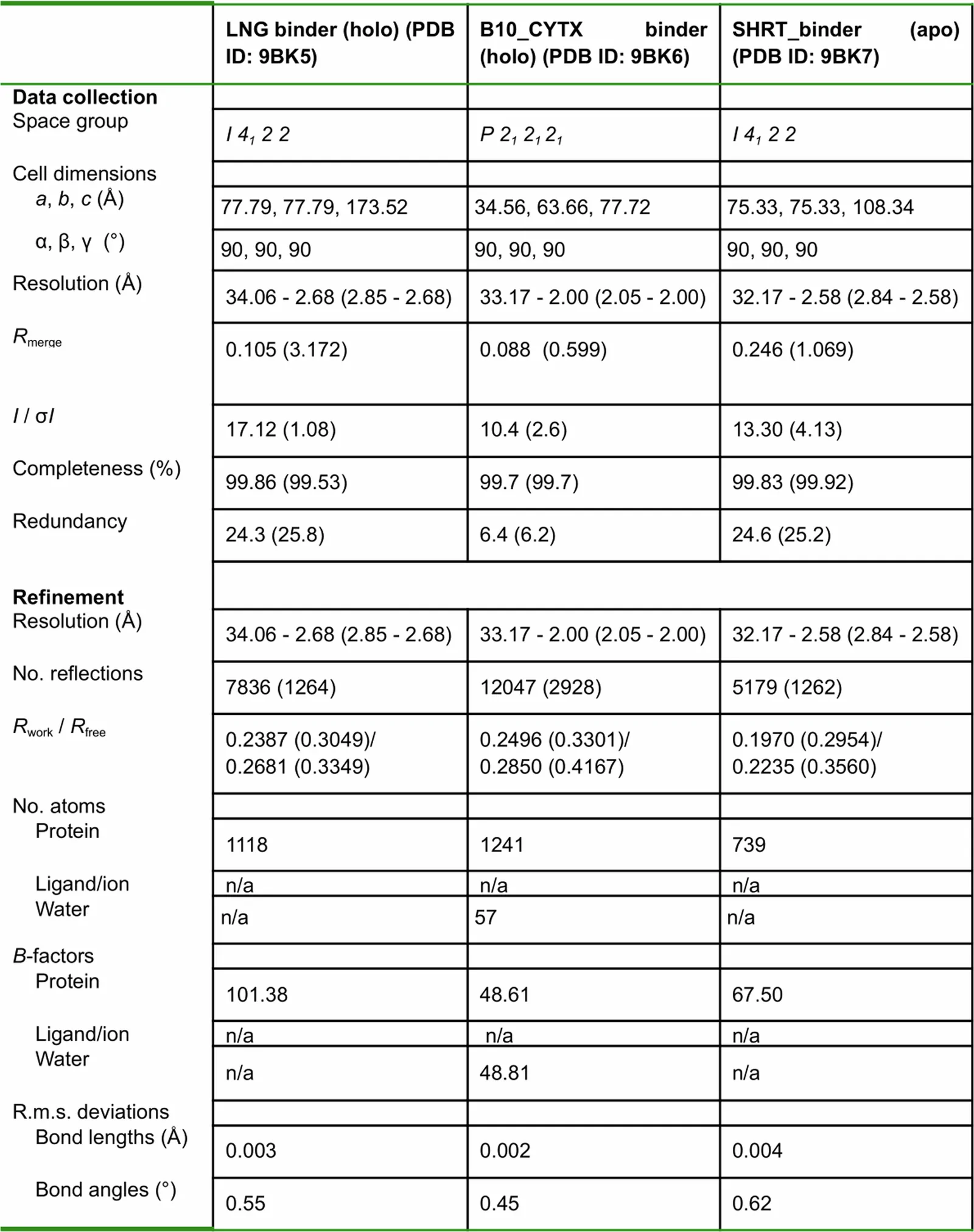
Data collection and refinement statistics (molecular replacement)

*Single Crystal used for each data collection. *Values in parentheses are for highest-resolution shell.

## Data Availability

Atomic models of the snake toxin binders have been deposited in the PDB. These include the apo-state structure of the SHRT design (PDB 9BK7) (Fig. 3a), the holo-state structure of the LNG design in complex with α-cobratoxin (PDB 9BK5) (Fig. 3b) and the CYTX_B10 design in complex with *N.* *pallida* cytotoxin (PDB 9BK6) (Fig. 3c).

## Code availability

Code explanation and examples of binder design using RFdiffusion can be found at https://github.com/RosettaCommons/RFdiffusion#binder-design.
