# Supplementary material for: De novo designed proteins neutralize lethal snake venom toxins
Source: Nature. 2025 Jan 15;639(8053):225–31. doi: 10.1038/s41586-024-08393-x (PMC11882462; doi:10.1038/s41586-024-08393-x)
Supplement: Supplementary file 1 [file 41586_2024_8393_MOESM1_ESM.pdf]

nature portfolio

https://doi.org/10.1038/s41586-024-08393-x

Supplementary information

De novo designed proteins neutralize lethal snake venom toxins

In the format provided by the authors and unedited

Supplementary Information: *De novo designed proteins neutralize lethal snake venom toxins*

Target	Binder ID	Binder amino acid sequence
ScNtx	SHRT	PKTVVVRLSPSMNEEQAAEIG REAGKAALAAGDRLVFVGPAD QSYAAMKAAMEAGLPEVTMYA LDFSDAESALKAAEVAEDEGD EEVAEVAREIAEEIKA
α-cobratoxin	LNG	SPAEREAGRIVVRGDVAIAEAV VRKVGEVAGKEVILLISYRKNG EWITYQRNLEATPEDVERTIAVI REIYEESGGDFILAIFSDPEVGA AGRAVAAAAA
Consensus cytotoxin	CYTX	SAEEKLKELEKKIEEFYKSAES VTSSISESNGTLTVTTTLKIGDA TVTATETYKIINGSPYKDGEVTY SASAEGKALADELAKLDKEYR EAQEKVEEEKK
Consensus cytotoxin	CYTX_B10	TPEERLAQLEKEIQALYDAADE VVDEVEEKDGKMTVTRTLTIGD GTVTLVETLKIVDGAPVKDGEIE VICNPECEELGKRLKALAKEYE KAQEEVEKAKA

Supplementary Table 1. Amino acid sequences of 3FTx binding proteins.

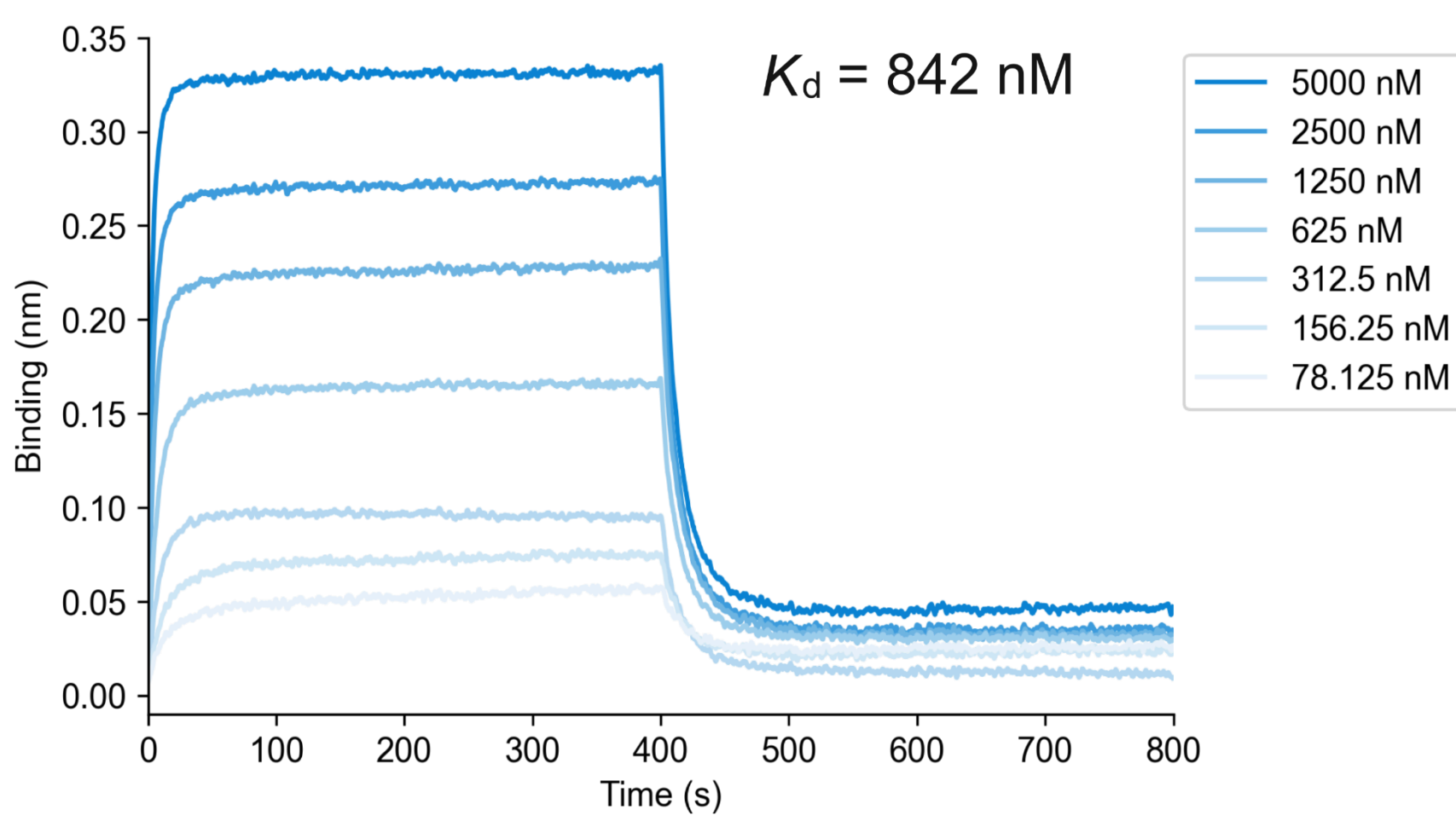

Figure S1. BLI traces of initial hit for ScNtx. Measurements indicate 842 nM binding affinity to ScNTx.

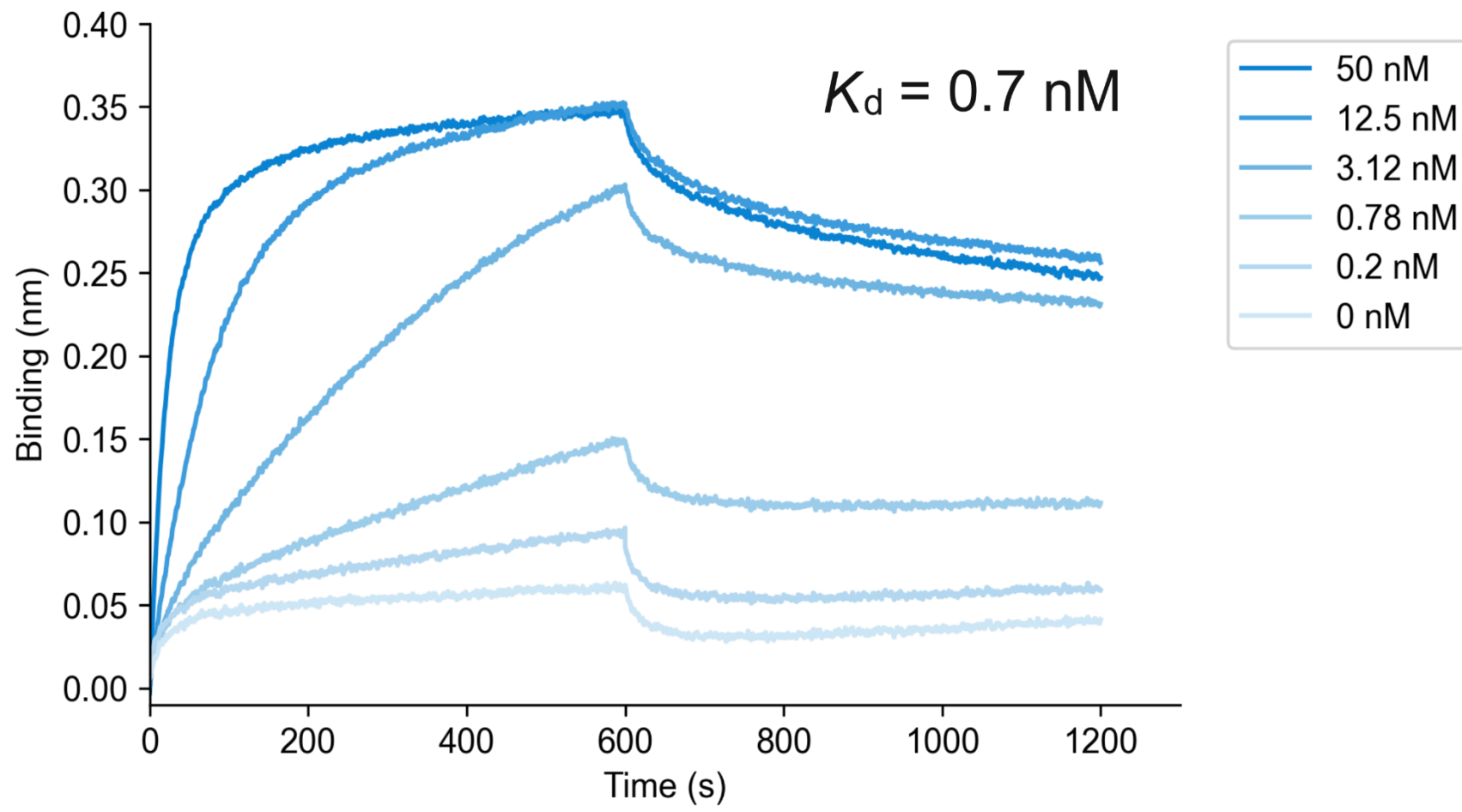

Figure S2. BLI traces of SHRT binder. Measurements indicate 0.7 nM binding affinity to ScNTx.

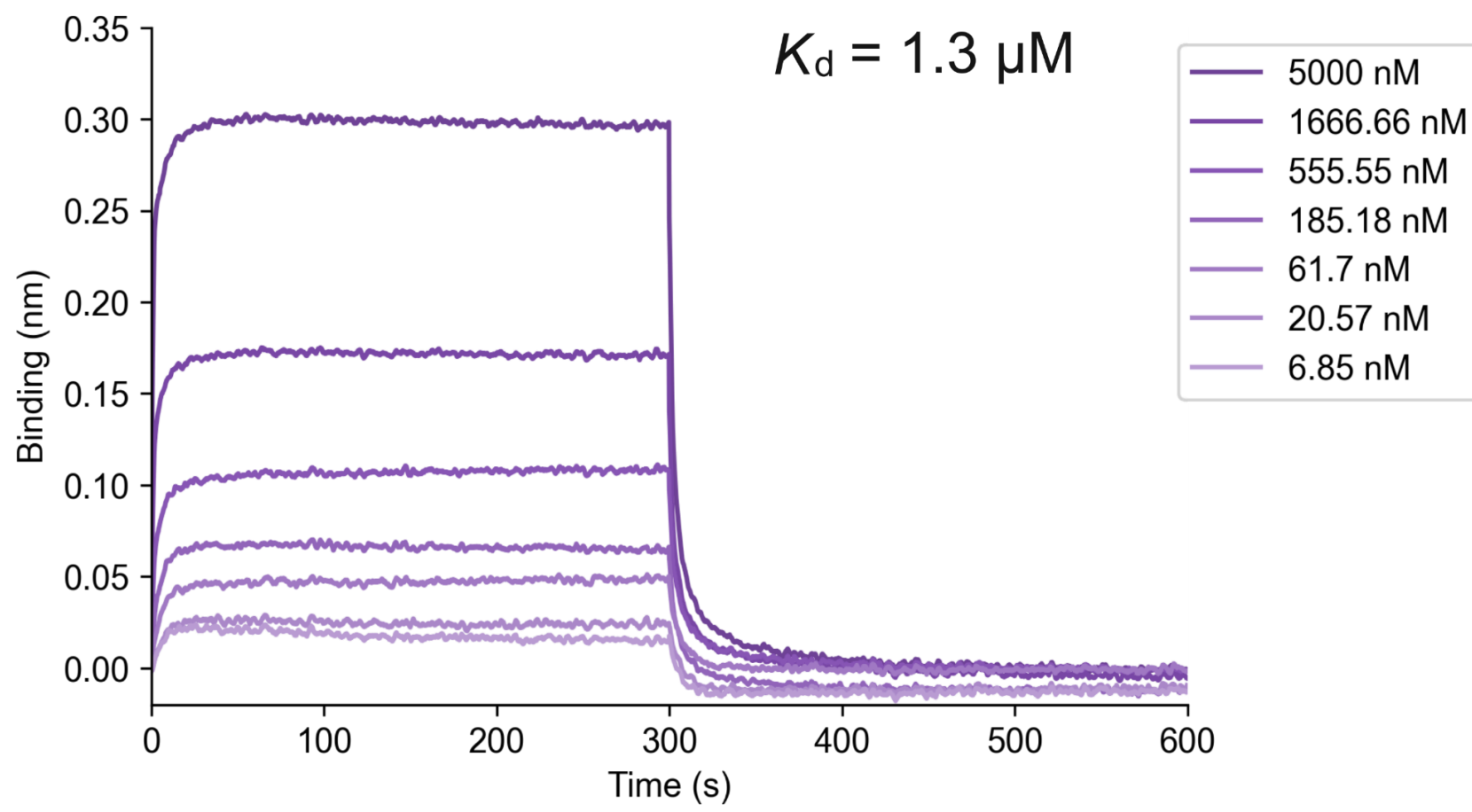

Figure S3. BLI traces of initial hit for α-cobratoxin. Measurements indicate 1.3 μM binding affinity to α-cobratoxin.

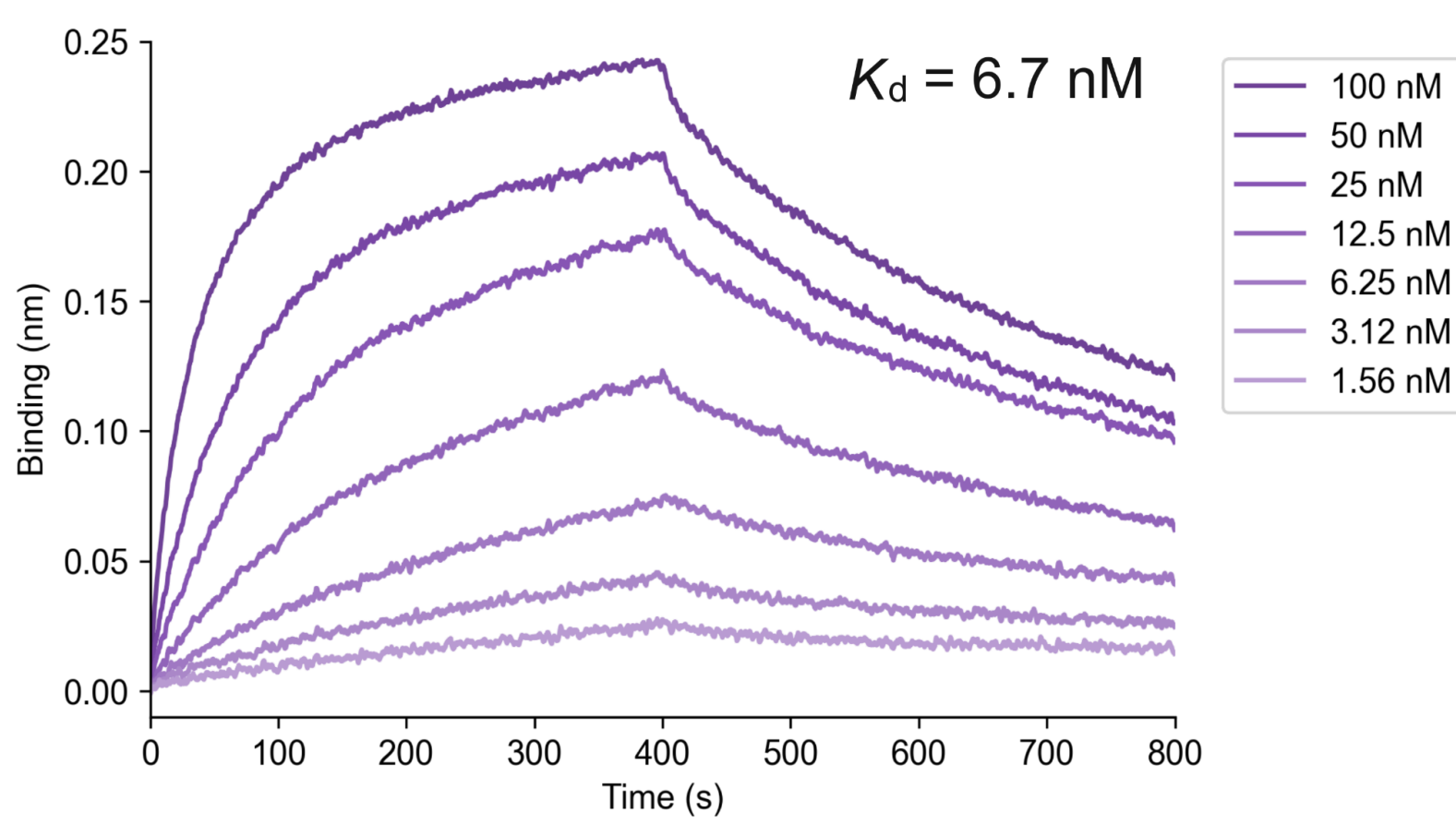

Figure S4. BLI traces of LNG binder. Measurements indicate 6.7 nM binding affinity to α-cobratoxin.

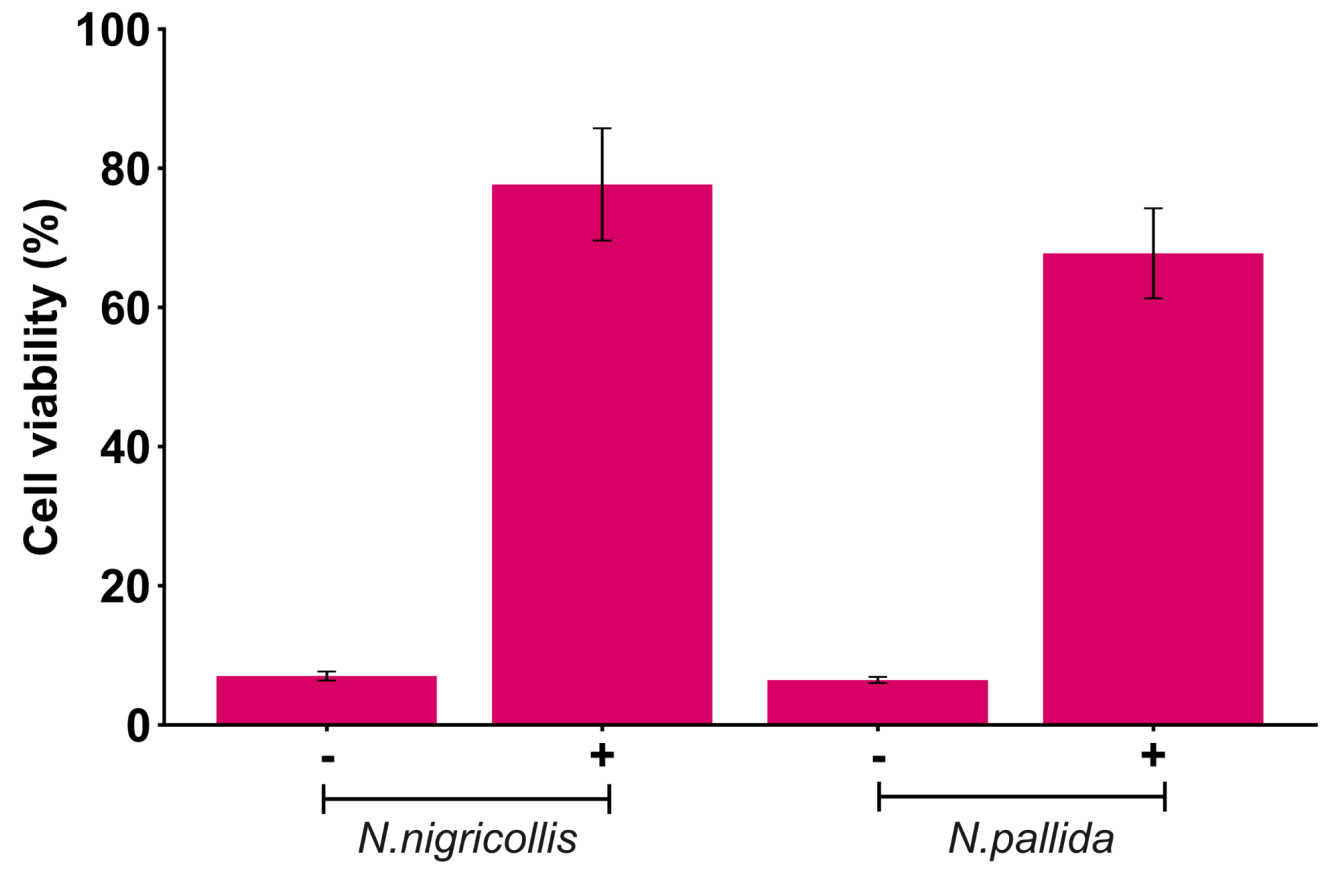

Figure S5. Initial screening of whole venoms for CYTX binder in HEK293T cells. Neutralization of the cytolytic effects of whole venoms from two different *Naja* species by the CYTX binder. Two IC_{50}s of the whole venoms were pre-incubated with the binder at a 1:5 molar ratio (toxin:binder).This ratio was estimated assuming 70% of the whole venom consists of cytotoxins based on prior proteomic analyses[1]. Experiments were performed in triplicates, and results are expressed as mean ± SD.

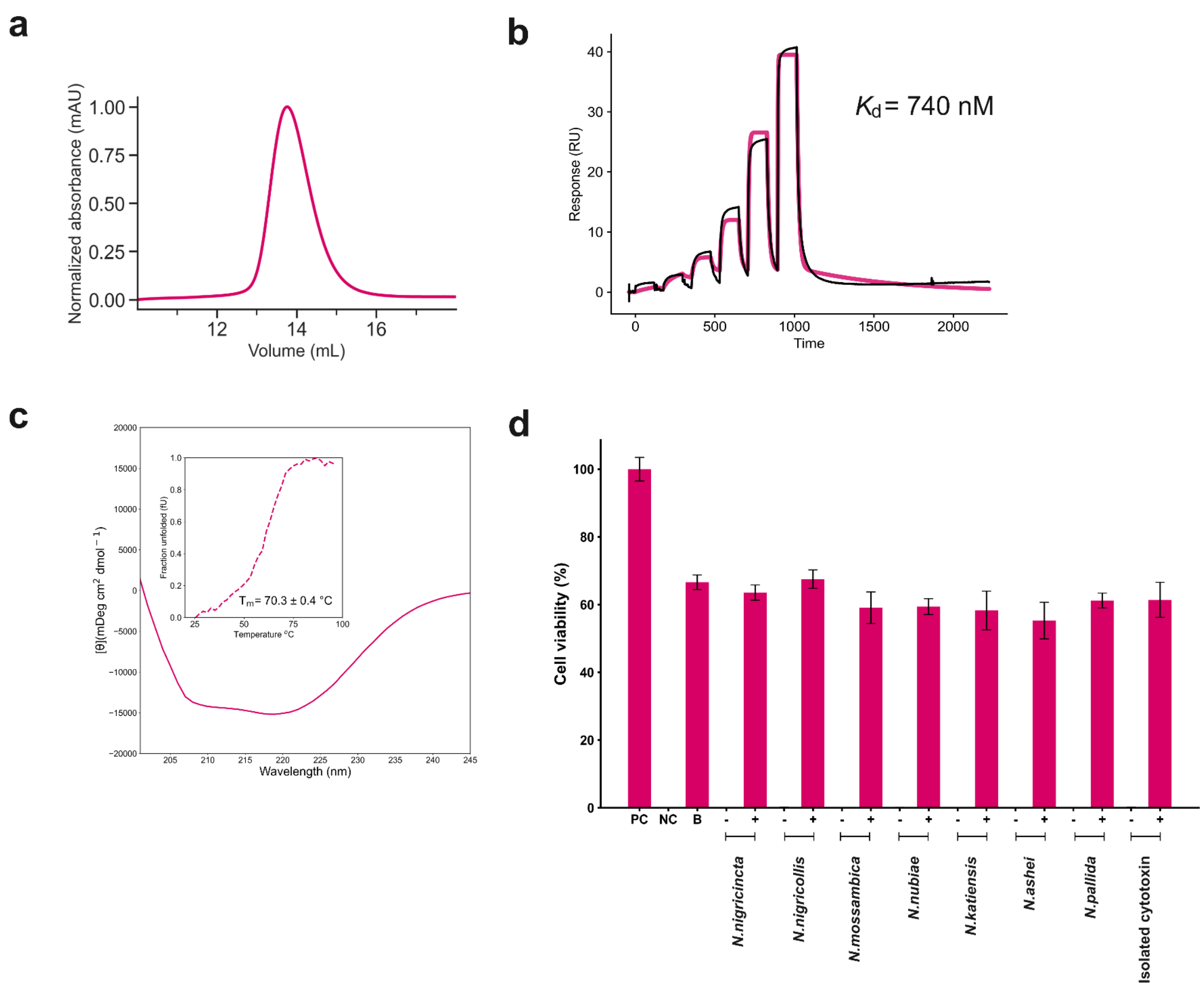

Figure S6. Experimental characterization of CYTX_B10 binder. (a) SEC trace of purified protein. **(b)** SPR binding affinity measurement. Colored solid line represent the fit using the heterogeneous ligand model, with dissociation constant (K_d) value derived from this fit**. (c)** CD data confirms the presence of

αβ-secondary structure in CYTX_B10 and its thermal stability (inset). **(d)** Neutralization of the cytolytic effects of whole venoms from seven different *Naja* species and isolated cytotoxin by the CYTX_B10 binder. Two IC_{50}s of the whole venoms or toxin were pre-incubated with CYTX_B10 at a 1:5 molar ratio (toxin:binder). This ratio was estimated assuming 70% of the whole venom consists of cytotoxins based on prior proteomic analyses[1]. Keratinocyte media was used as a positive control (PC). Triton X-100 was used as a negative control (NC). CYTX_B10 binder (B) was used as a positive control. (-) denotes two IC_{50}s of the whole venoms without binder, and (+) denotes venoms incubated with binder. Experiments were performed in triplicates, and results are expressed as mean ± SD.

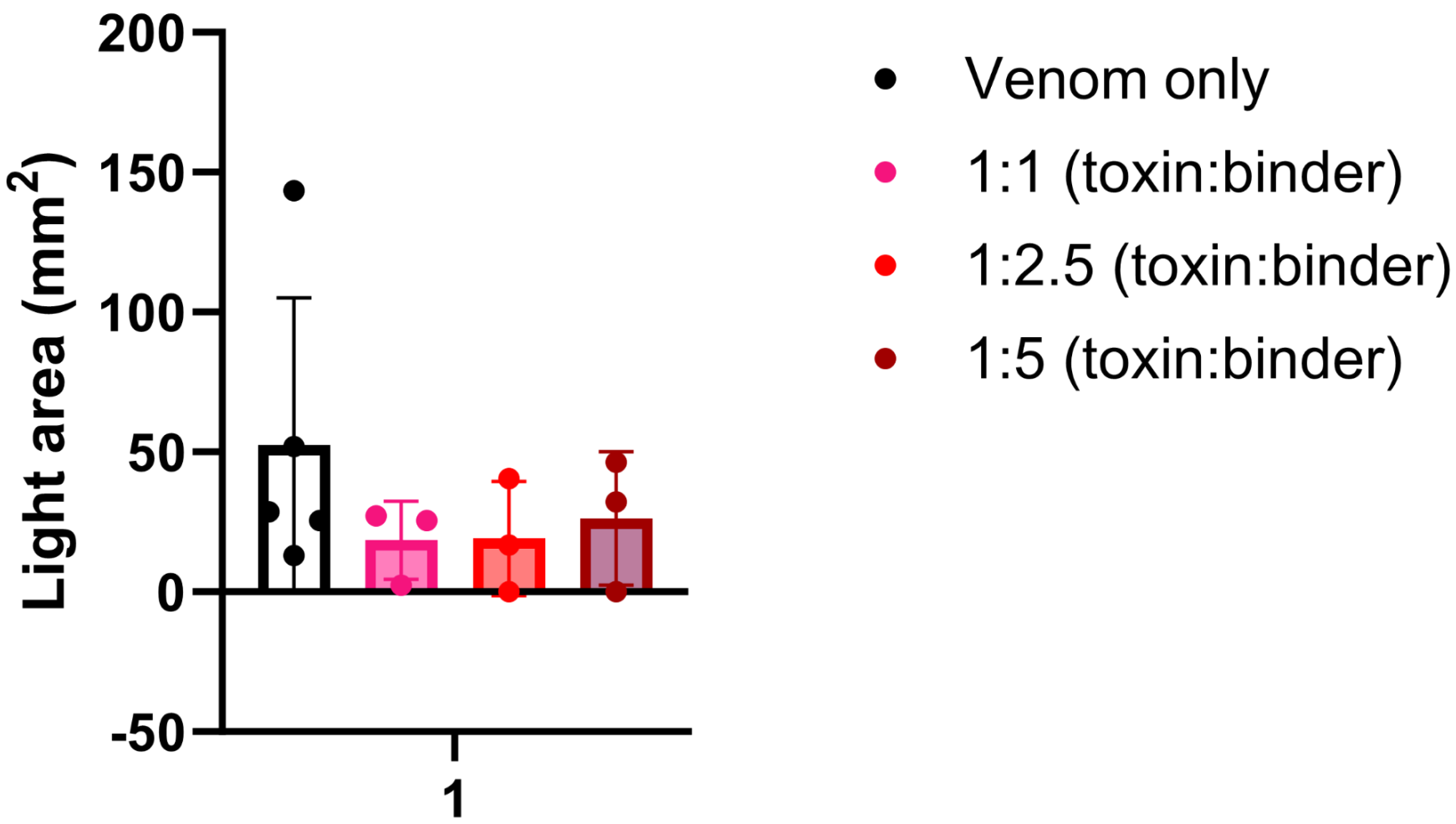

Figure S7. Histopathological analysis of light lesions induced by West African *N. nigricollis* whole venom by VIDAL[2]. Lesions induced by the whole venom, preincubated with varying concentrations of CYTX binder, do not exhibit a significant reduction in size. An outlier within the venom-only group is observed, likely attributable to biological variation.

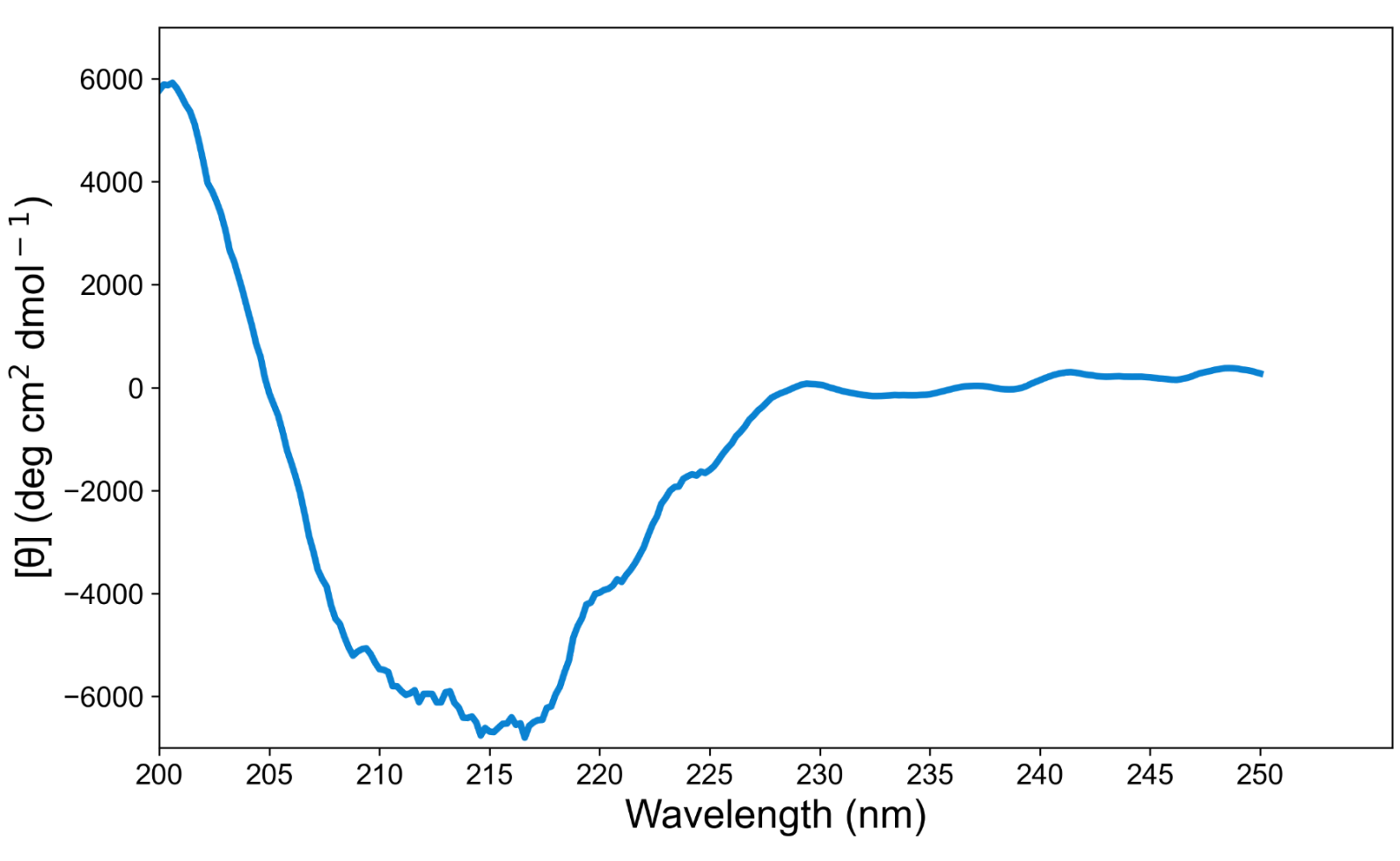

Figure S8. CD spectra of ScNtx. CD data confirms the presence of β-secondary structure of the recombinantly expressed toxin.

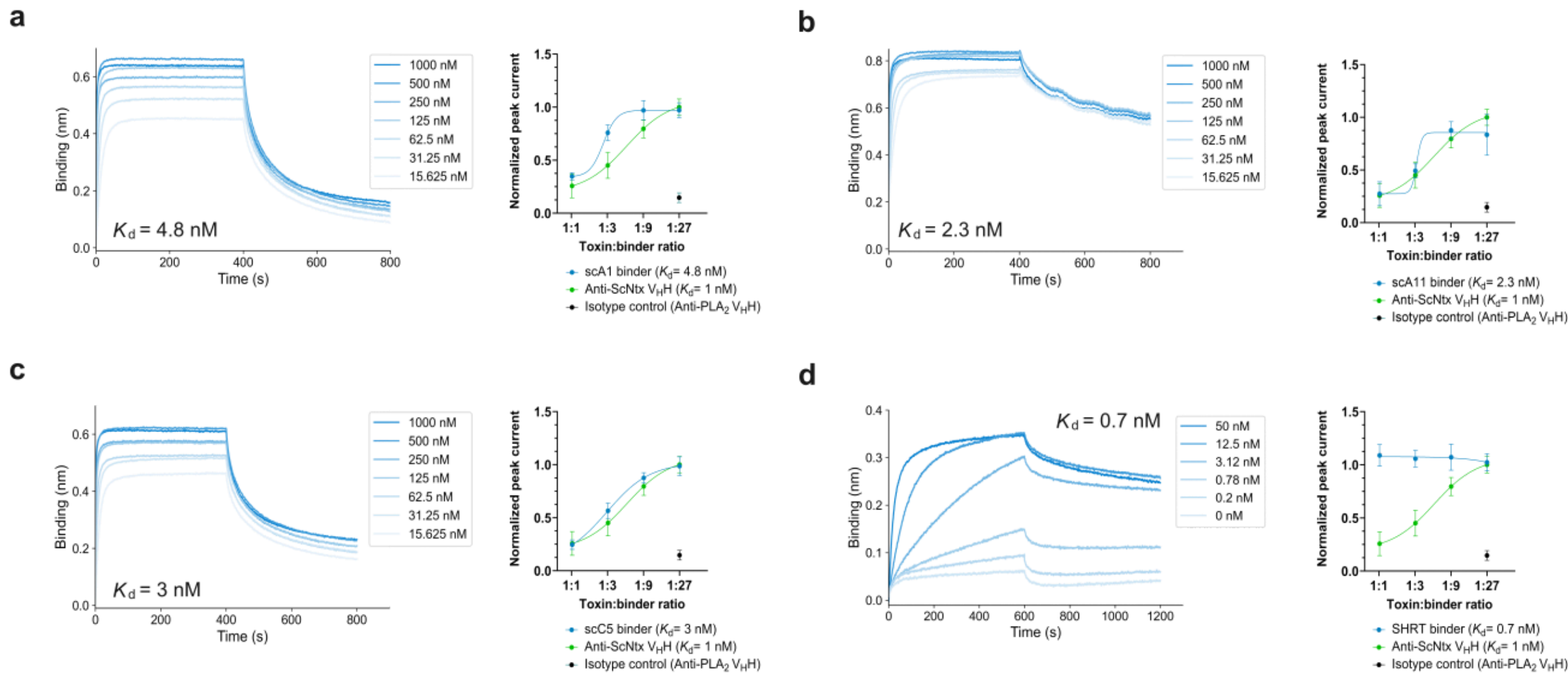

Figure S9. BLI traces and patch-clamp functional neutralization of top ScNtx binding proteins. (a) scA1 binder. Left: BLI measurements indicate 4.8 nM binding affinity to ScNTx. Right: concentration-response curves comparing the efficacy of scA1 binder and anti-ScNtx V_HH in preventing nAChR blocking by one IC_{80} of ScNtx. **(b)** scA11 binder. Left: BLI measurements indicate 2.3 nM binding affinity to ScNTx. Right: concentration-response curves comparing the efficacy of scA11 binder and anti-ScNtx V_HH in preventing nAChR blocking by one IC_{80} of ScNtx. **(c)** scC5 binder. Left: BLI measurements indicate 3 nM binding affinity to ScNTx. Right: concentration-response curves comparing the efficacy of scC5 binder and anti-ScNtx V_HH in preventing nAChR blocking by one IC_{80} of ScNtx. **(d)** SHRT binder. Left: BLI measurements indicate 0.7 nM binding affinity to ScNTx. Right: concentration-response curves comparing the efficacy of SHRT binder and anti-ScNtx V_HH in preventing nAChR blocking by one IC_{80} of ScNtx.

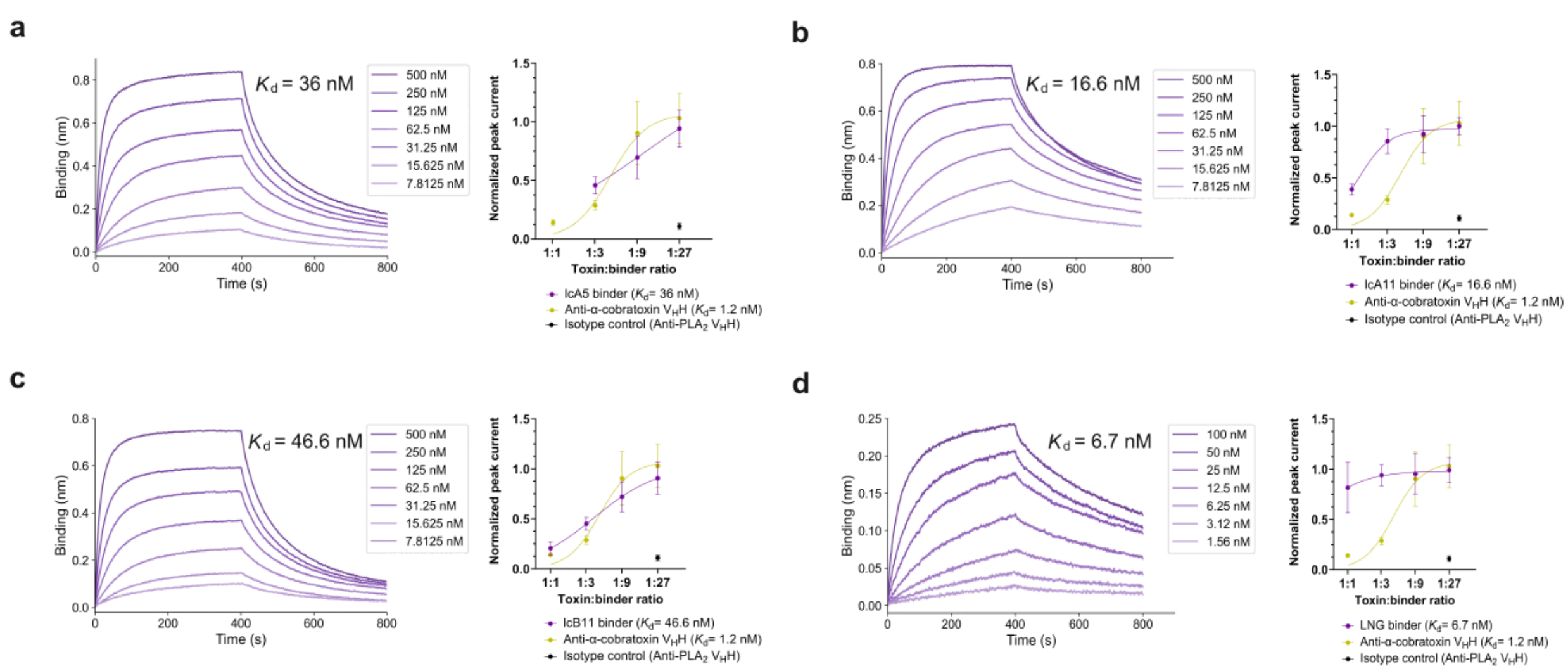

Figure S10. BLI traces and patch-clamp functional neutralization of top α-cobratoxin binding proteins. (a) IcA5 binder. Left: BLI measurements indicate 36 nM binding affinity to α-cobratoxin. Right:concentration-response curves comparing the efficacy of IcA5 binder and anti-α-cobratoxin V_HH in preventing nAChR blocking by one IC_{80} of α-cobratoxin. **(b)** IcA11 binder. Left: BLI measurements indicate 16.6 nM binding affinity to α-cobratoxin. Right: concentration-response curves comparing the efficacy of

lcA11 binder and anti-α-cobratoxin V_HH in preventing nAChR blocking by one IC_{80} of α-cobratoxin. **(c)** lcB11 binder. Left: BLI measurements indicate 46.6 nM binding affinity to α-cobratoxin. Right: concentration-response curves comparing the efficacy of lcB11 binder and anti-α-cobratoxin V_HH in preventing nAChR blocking by one IC_{80} of α-cobratoxin. **(d)** LNG binder. Left: BLI measurements indicate 6.7 nM binding affinity to α-cobratoxin. Right: concentration-response curves comparing the efficacy of LNG binder and anti-α-cobratoxin V_HH in preventing nAChR blocking by one IC_{80} of α-cobratoxin.

References:

1.Petras, D. *et al.* Snake Venomics of African Spitting Cobras: Toxin Composition and Assessment of Congeneric Cross-Reactivity of the Pan-African EchiTAb-Plus-ICP Antivenom by Antivenomics and Neutralization Approaches. *J. Proteome Res.* **10**, 1266–1280 (2011).
2.Laprade, W. *et al.* Machine-learning guided Venom Induced Dermonecrosis Analysis tooL: VIDAL. *Sci. Rep.* **13**, 21662 (2023).
