# Supplementary material for: De novo designed proteins neutralize lethal snake venom toxins
Source: Nature. 2025 Jan 15;639(8053):225–31. doi: 10.1038/s41586-024-08393-x (PMC11882462; doi:10.1038/s41586-024-08393-x)
Supplement: Supplementary file 2 [file 41586_2024_8393_MOESM2_ESM.pdf]

nature portfolio

Corresponding author(s): David Baker
Last updated by author(s): 2024/10/02

Reporting Summary

Nature Portfolio wishes to improve the reproducibility of the work that we publish. This form provides structure for consistency and transparency in reporting. For further information on Nature Portfolio policies, see our Editorial Policies and the Editorial Policy Checklist.

Statistics

For all statistical analyses, confirm that the following items are present in the figure legend, table legend, main text, or Methods section.

n/a	Confirmed	
☐	☒	The exact sample size (*n*) for each experimental group/condition, given as a discrete number and unit of measurement
☐	☒	A statement on whether measurements were taken from distinct samples or whether the same sample was measured repeatedly
☒	☐	The statistical test(s) used AND whether they are one- or two-sided<br>*Only common tests should be described solely by name; describe more complex techniques in the Methods section.*
☒	☐	A description of all covariates tested
☒	☐	A description of any assumptions or corrections, such as tests of normality and adjustment for multiple comparisons
☐	☒	A full description of the statistical parameters including central tendency (e.g. means) or other basic estimates (e.g. regression coefficient) AND variation (e.g. standard deviation) or associated estimates of uncertainty (e.g. confidence intervals)
☒	☐	For null hypothesis testing, the test statistic (e.g. *F*, *t*, *r*) with confidence intervals, effect sizes, degrees of freedom and *P* value noted<br>*Give P values as exact values whenever suitable.*
☒	☐	For Bayesian analysis, information on the choice of priors and Markov chain Monte Carlo settings
☒	☐	For hierarchical and complex designs, identification of the appropriate level for tests and full reporting of outcomes
☒	☐	Estimates of effect sizes (e.g. Cohen's *d*, Pearson's *r*), indicating how they were calculated

Our web collection on statistics for biologists contains articles on many of the points above.

Software and code

Policy information about availability of computer code

Data collection	RFdiffusion 1.0.0, AlphaFold2, ,ProteinMPNN, CCP4i2 program suite
Data analysis	Matplotlib 3.6.2, SciPy 1.9.3, Seaborn 0.11.2, PyMOL 2.5.0,Phaser,COOT Gtk+2: 0.9.8.95,Sophion Analyzer v6.6.70,GraphPad Prism v10.1.1, VIDAL1

For manuscripts utilizing custom algorithms or software that are central to the research but not yet described in published literature, software must be made available to editors and reviewers. We strongly encourage code deposition in a community repository (e.g. GitHub). See the Nature Portfolio guidelines for submitting code & software for further information.

Data

Policy information about availability of data

All manuscripts must include a data availability statement. This statement should provide the following information, where applicable:

- Accession codes, unique identifiers, or web links for publicly available datasets
- A description of any restrictions on data availability
- For clinical datasets or third party data, please ensure that the statement adheres to our policy

Atomic models of the snake toxin binders have been deposited in the Protein Data Bank. These include the apo-state structure of the SHRT design (PDB ID: 9BK7, Fig. 3a), the holo-state structure of the LNG design in complex with α-cobratoxin (PDB ID: 9BK5, Fig. 3b), and the CYTX_B10 design in complex with Naja pallida cytotoxin (PDB ID: 9BK6, Fig. 3c).

Field-specific reporting

Please select the one below that is the best fit for your research. If you are not sure, read the appropriate sections before making your selection.

☒ Life sciences ☐ Behavioural & social sciences ☐ Ecological, evolutionary & environmental sciences

For a reference copy of the document with all sections, see nature.com/documents/nr-reporting-summary-flat.pdf

Life sciences study design

All studies must disclose on these points even when the disclosure is negative.

Sample size	We used three animals per dose to determine LD50 values, minimizing animal use while demonstrating proof-of-concept for neutralizing snake venom toxins with our de novo protein binders. Determining precise LD50 values is not relevant here, as our proteins are experimental. In our in vivo protection assay, we used five mice. A small-scale trial with a well-defined endpoint, such as overall mortality, effectively tests preclinical hypotheses and demonstrates the therapeutic potential of our binders to neutralize lethal snake toxin
Data exclusions	None
Replication	Each dataset contains many (n reported in figure legends) independent measurements.
Randomization	Snake venom has a well-defined, immediate toxic effect, and antivenom neutralization follows a clear biological pathway. Because the biological response to venom and antivenom is relatively predictable, randomization may not add significant value to the validity of the experiment
Blinding	N/A (all analysis was automated)

Reporting for specific materials, systems and methods

We require information from authors about some types of materials, experimental systems and methods used in many studies. Here, indicate whether each material, system or method listed is relevant to your study. If you are not sure if a list item applies to your research, read the appropriate section before selecting a response.

Materials & experimental systems

n/a	Involved in the study	
☒	☐	Antibodies
☐	☒	Eukaryotic cell lines
☒	☐	Palaeontology and archaeology
☐	☒	Animals and other organisms
☒	☐	Human research participants
☒	☐	Clinical data
☒	☐	Dual use research of concern

Methods

n/a	Involved in the study	
☒	☐	ChIP-seq
☒	☐	Flow cytometry
☒	☐	MRI-based neuroimaging

Eukaryotic cell lines

Policy information about cell lines

Cell line source(s)	HEK293T were obtained from ATCC, N/TERT immortalized keratinocytes were kindly provided by Edel O'Toole from the Queen Mary University of London
Authentication	Morphology check by microscope
Mycoplasma contamination	All cell lines tested negative for mycoplasma contamination
Commonly misidentified lines (See ICLAC register)	N/A

Animals and other organisms

Policy information about studies involving animals; ARRIVE guidelines recommended for reporting animal research

Laboratory animals	NSA CD1 male mice (Charles River, 18-20 g)
Wild animals	No wild animals were used in the study

Field-collected samples	N/A
Ethics oversight	Animal experiments for in vivo neurotoxicity assays were conducted at the University of Northern Colorado under protocol 2303D-SM-S-26, approved by the UNC Institutional Animal Care and Use Committee (UNC-IACUC), in accordance with Government Principles, Public Health Policy, USDA Animal Welfare Act, and the Guide for the Care and Use of Laboratory Animals.<br><br>Animal experiments for in vivo dermonecrosis assays were approved by the Animal Welfare and Ethical Review Boards of the Liverpool School of Tropical Medicine and the University of Liverpool, and conducted under UK Home Office project license P58464F90 in accordance with the UK Animal (Scientific Procedures) Act 1986.

Note that full information on the approval of the study protocol must also be provided in the manuscript.
