# Supplementary material for: De novo designed proteins neutralize lethal snake venom toxins
Source: Nature. 2025 Jan 15;639(8053):225–31. doi: 10.1038/s41586-024-08393-x (PMC11882462; doi:10.1038/s41586-024-08393-x)
Supplement: Supplementary file 3 [file 41586_2024_8393_MOESM3_ESM.pdf]

nature portfolio

Peer Review File

De novo designed proteins neutralize lethal snake venom toxins

Corresponding Author: Professor David Baker

This file contains all reviewer reports in order by version, followed by all author rebuttals in order by version.

Version 0:

Reviewer comments:

Referee #1

(Remarks to the Author)
This study demonstrates the usefulness of deep-learning methods in designing neutralizing proteins against snake venoms.

• How was the structure of recombinantly expressed ScNtx validated? The manuscript mentions that only SDS-PAGE was used to verify the molecular weight. How did the authors validate the folding of the toxin?

• Why were only 3 mice used in the animal experiments? This is a non-standard practice and needs justification. Even the weights of the mice (20-30 g) were non-standard.

• The methods section relating to the in vivo section is unclear. Did the authors follow the ip route of venom-binder administration for the preincubation experiment? If yes, then why didn't they use the iv route, which is more appropriate for this experiment?

• "We chose to target our design efforts against the neurotoxin edge β-strands (previously discovered monoclonal antibodies in contrast mimic the nAChR binding site14,21,22), focusing on binding modes blocking neurotoxin binding to nAChRs through steric hindrance."
It would be helpful to the readers to know why this strategy was followed.

• Why wasn't a consensus sequence constructed for long chain α-cobratoxin? It would help the reader understand the rationale.

• The exact interaction modality between the protein inhibitor and long-chain 3FTx is mentioned. However, no such clarity is presented for short-chain 3FTx.

• "fetal bovine serum at 37 °C and 5% and CO_2."
The second 'and' should be deleted.

Referee #2

(Remarks to the Author)
This paper is about using recent protein design tools to design proteins that can neutralize snake venom toxins.
Overall from a biology point of view the paper seems like a very solid contribution to a very important problem, although I should preface this that this area is outside my general area of expertise, so the rest of the review will focus on the design methodology itself and not discuss the experimental validation, as other reviewers will be better placed to judge this.

The methodology uses RFDiffusion for designing the backbone, then uses ProteinMPNN to design the sequence and after filters designs using AlphaFold2 and Rosettafold.
In fact this methodology is where one of my main criticisms of the paper comes in, the last sentence in the abstract is:

“Beyond snakebite, our computational design methodology should help democratize therapeutic discovery, particularly in resource-limited settings, by substantially reducing costs and resource requirements for development of therapies to neglected tropical diseases.”
Which suggests that the paper claims that it is introducing a computational design methodology, however as far as I can tell this is just applying the methodology of the RFDiffusion publication to a new domain and as such I think there’s no new methodology.
If there is a new methodology the authors should point out more carefully what they think is new about this methodology in the paper, otherwise the language in the abstract should be toned down to make clear that the paper doesn’t propose a new methodology, parts of the main section are more clear that it’s just using the method set out in RFDiffusion.

In general snake venom typically isn’t a single protein, but rather a cocktail of a very large number of different toxic components. I think it would be useful if the authors could address why targeting a single protein is effective here.
Also I wasn’t entirely clear on the “In vivo protection” section, is this testing efficacy of the designed protein against the particular compound it was designed for or testing efficacy against what would be the usecase, i.e. the snake venom which contains many other compounds. If it’s the former I think this should be spelled out more clearly. Also how do the authors see this turning into a therapeutic? Would there be a need to design separate binders and manufacture them for each component in the venom?

Aside from this the paper is very well written and easy to follow. The experimental validation of the designs looks very solid to me. However, as this paper is about applying RFDiffusion to designing binders of a particular component of snake venom, whether this paper is suitable for publication in Nature rests entirely on the application itself, as I’m not an expert on snake venoms I’d refer to other reviewers for this.

Referee #3

(Remarks to the Author)
The article by David Baker and co-workers elegantly presents the de novo design of proteins that neutralise snake venom 3FTXs. These toxins are very difficult to neutralise with animal-derived antibodies and are central to the pathophysiology of elapid snake envenomation. As this snake family claims tens of thousands of lives annually, the problem is timely and very relevant.
The study is very sound; the data is solid and robustly supports the conclusions. The work is groundbreaking - as far as I know, this is the first time de novo proteins have been designed to neutralise snake venom components. As such, this work opens up a whole new field of research in the agonising effort to help snakebite victims.
In summary, I have no doubt that this fantastic research is worthy of publication in Nature with minor changes. As a computational biochemist, I will not go into the more experimental aspects but will focus on the structural data and their fundamental significance.

1. Page 3: The authors state that " Synthetic genes encoding 44 designs targeting ScNTx were screened via yeast surface display (YSD), and one candidate was identified to bind to ScNTx with a dissociation constant (Kd) of 842 nM (…)".
The reader would like to know the success rate of the method. Was this candidate protein the only submicromolar binder? Were there other protein candidates with similar affinities? The same question applies to affinity optimisation. What was the distribution of affinities among the 78 candidates of which one had an affinity of 0.9 nM?
2. In the following paragraph, the authors describe the design of neutralising proteins against the monoclonal cobra 3FTxs. It is not immediately clear why they decided to do this after designing a neutralising protein for the consensus sequence toxin (although it becomes clear after seeing the results). A sentence stating the aims of this experiment would be useful here.
3. Does the monocled cobra venom have multiple alpha-neurotoxin isoforms or a single isoform? A UniProt ID would help to unambiguously define the alpha-neurotoxin sequence.
4. Still on the same page, the authors state: "An arginine residue at position 33, located at the tip of loop II of α-cobratoxin, makes extensive electrostatic interactions with the binder".
Do the authors have evidence that these interactions are important for affinity? Although Arg residues are often found in protein-protein binding hotspots, the Arg33 solvation free energy in the dissociated state may compensate for the binding free energy in the bound state. Therefore, establishing strong intermonomer interactions does not necessarily imply a positive contribution to affinity.
5. Page 4, first sentence - is the reference to Figure 1d correct?
6. It would be interesting to retain the acetylcholine shown in Figures 1c and 1d, perhaps in a transparent material.
7. The mode of action of the antibodies is not immediately clear to me, especially after looking at Fig 1d. If the neutralizing proteins mimic the toxins, why are they not toxic themselves? How can acetylcholine bind and open the receptor in the presence of the bound neutralizing protein?
8. Why do the CYTXs have a much lower affinity than previously designed neutralizing proteins?
9. Finally, it seems that obtaining interspecific neutralizing proteins by targeting the toxins rather than mimicking the receptors will be a challenge. What do the authors have to comment about this?

Version 1:

Reviewer comments:

Referee #1

(Remarks to the Author)
The authors have sufficiently addressed all my comments, and I am happy with the revised version of the manuscript. I recommend the publication of this very exciting, well-planned, and well-executed study in Nature.

Referee #2

(Remarks to the Author)
I want to thank the authors for the detailed feedback.
The authors addressed all my questions satisfactorily.

Referee #3

(Remarks to the Author)
The authors have addressed all my questions and comments except one, which still puzzles me.

Still on the mechanism of action of the neutralizing proteins - if they block acetylcholine binding, as shown in Figure 1c, why don't they inhibit nicotinic acetylcholine receptors and display the same neurotoxic effect as the snake toxins?

Apart from that, this excellent scientific paper is ready for publication. It fully meets Nature's strict criteria for quality and impact.

Open Access This Peer Review File is licensed under a Creative Commons Attribution 4.0 International License, which permits use, sharing, adaptation, distribution and reproduction in any medium or format, as long as you give appropriate credit to the original author(s) and the source, provide a link to the Creative Commons license, and indicate if changes were made.
In cases where reviewers are anonymous, credit should be given to 'Anonymous Referee' and the source.
The images or other third party material in this Peer Review File are included in the article's Creative Commons license, unless indicated otherwise in a credit line to the material. If material is not included in the article's Creative Commons license and your intended use is not permitted by statutory regulation or exceeds the permitted use, you will need to obtain permission directly from the copyright holder.
To view a copy of this license, visit https://creativecommons.org/licenses/by/4.0/

Editor:

Your manuscript, "De novo designed proteins neutralize lethal snake venom toxins", has now been seen by 3 referees, whose comments are attached below. While they find your work of potential interest, as do we, they have raised important concerns that in our view need to be addressed before we can consider publication in Nature.

The referees have convergent issues about the need to revise the framing of the study, and they feel that in order to be suited for publication, extensive additional validation would be required to support the applicability in the context of complex toxins, and show robust in vivo efficacy.

Should further experiments allow you to address these criticisms, we would be happy to consider a revised manuscript (unless something similar has been accepted at Nature or appeared elsewhere in the meantime). We hope to receive your revised paper within four to six months. If you cannot complete the required revisions within this time frame, please let us know when you would anticipate being able to submit a revised manuscript. We also strongly suggest that your revised manuscript has tracked changes, which is increasingly requested by referees to aid in their re-review.

Response: We have significantly expanded and clarified the original manuscript to address the constructive criticisms raised by the referees. Specifically, we have added detailed explanations of the design rationale and experimental results throughout the manuscript.

We have incorporated a circular dichroism spectrum of the recombinantly expressed short-chain α-neurotoxin (ScNtx) to validate its proper folding (Supplementary Fig. S8). Additionally, we have included more experimental details regarding the success rate of our design method in Supplementary Figures S9 and S10.

We believe that the revised manuscript clearly stands on its own as a full paper in Nature.

Referee #1 (Remarks to the Author):

This study demonstrates the usefulness of deep-learning methods in designing neutralizing proteins against snake venoms.

Response: The authors would like to thank the reviewer for taking the time to go through this manuscript and provide valuable feedback.

• How was the structure of recombinantly expressed ScNtx validated? The manuscript mentions that only SDS-PAGE was used to verify the molecular weight. How did the authors validate the folding of the toxin?

Response: The reviewer is correct in that SDS-PAGE was used to confirm the molecular weight. We have now included a circular dichroism spectrum of the recombinantly expressed ScNtx in Supplementary Fig. S8. This confirms its beta-sheet-rich structure and validates its proper folding. The functional activity of ScNtx is further demonstrated by patch clamp (Fig.4a) and *in vivo* experiments (Fig.4d).

• Why were only 3 mice used in the animal experiments? This is a non-standard practice and needs justification. Even the weights of the mice (20-30 g) were non-standard.

Response: We used three animals per dose to determine LD_{50} values, minimizing animal use while demonstrating proof-of-concept for neutralizing snake venom toxins with our *de novo* protein binders**.** Determining precise LD_{50} values is not relevant here, as our proteins are experimental. Unlike other studies using 60-100 animals[1,2], our approach involves ~15 animals in total, yielding robust and reproducible results. Expressing LD_{50}s to 2-3 decimal places is biologically irrelevant due to variations in venom composition and demographic factors, which affect the accuracy and utility of toxicity measures[3,4]. In our *in vivo* protection assay, we used five mice. A small-scale trial with a well-defined endpoint, such as overall

mortality, effectively tests preclinical hypotheses and demonstrates the therapeutic potential of our binders to neutralize lethal snake toxins[5]. Our results, shown in Fig. 4d, highlight the reproducibility of our binders in protecting mice from lethal neurotoxin challenges across different administration methods (pre-incubation and rescue assays) and toxin:binder ratios.

NSA mice weighing 20-30 g, categorized as young male adults (4-6 weeks old) are standard, as shown in https://www.inotivco.com/model/hsd-nsa-cf-1 (Check growth curve). This methodology has been validated and published previously[4].

References

1. Reed, L. J. & Muench, H. A SIMPLE METHOD OF ESTIMATING FIFTY PERCENT ENDPOINTS12. *Am. J. Epidemiol.* **27**, 493–497 (1938).
2. Kondo, H. *et al.* STANDARDIZATION OF ANTIVENINE I. A METHOD FOR DETERMINATION OF ANTILETHAL POTENCY OF HABU ANTIVENINE. *Jpn. J. Med. Sci. Biol.* **18**, 101–110 (1965).
3. Smith, C. F. *et al.* The best of both worlds? Rattlesnake hybrid zones generate complex combinations of divergent venom phenotypes that retain high toxicity. *Biochimie* **213**, 176–189 (2023).
4. Mackessy, S. P. *et al.* Venomics of the Central American Lyre Snake Trimorphodon quadruplex (Colubridae: Smith, 1941) from Costa Rica. *J. Proteomics* **220**, 103778 (2020).
5. Colin, L. & Smith, B. Statistical Considerations in Proof-of-Concept Studies. in *Statistical Methods in Biomarker and Early Clinical Development* (eds. Fang, L. & Su, C.) 221–245 (Springer International Publishing, Cham, 2019). doi:10.1007/978-3-030-31503-0_11.

• The methods section relating to the in vivo section is unclear. Did the authors follow the ip route of venom-binder administration for the preincubation experiment? If yes, then why didn't they use the iv route, which is more appropriate for this experiment?

Response: We have revised the methods section of the *in vivo* neurotoxicity assays. As suggested by the reviewer, we clarified the use of the intraperitoneal (IP) route for the preincubation experiment. The IP route is routinely used for toxicity assays[1,2], and our results showed that the IP toxicity of purified toxins was comparable to published intravenous (IV) values[3,4]. Switching to the IV route for venom/toxin challenge in rescue experiments is impractical due to the rapid envenoming it causes (leaving no therapeutic window to measure rescue) and is not biologically realistic, as IV injection during snakebites is rare[5]. For this reason, rescue experiments described in the literature do not use an intravenous dosing route for venom challenge[6-8]. While we could have delivered the binders via the intravenous route post-venom challenge, for these experiments we preferred to deliver the rescue to the same location as toxin injection, and thus used the IP route.

The modified text is shown below:

***In vivo* neurotoxicity protein binder protection assays section**

"In pre-incubation experiments, three LD_{50}s of the toxins (α-cobratoxin: 0.294 μg/g mouse; ScNtx: 0.261 μg/g mouse) were mixed with a 10-fold molar excess of their respective protein binders in PBS and incubated at room temperature for 30 minutes before intraperitoneal (i.p.) administration. Groups of five mice were injected with the binder:toxin mixture and observed at 2 and 24 hours. In rescue experiments, toxins (3 LD_{50}s) were administered i.p. 15 or 30 minutes before the corresponding binder, given i.p. at either 10- or 5-fold molar excess to groups of five mice. Protection against lethality was measured as percent mortality at 24 hours".

References

1. Smith, C. F. *et al.* The best of both worlds? Rattlesnake hybrid zones generate complex combinations of divergent venom phenotypes that retain high toxicity. *Biochimie* **213**, 176–189 (2023).
2. Mackessy, S. P. *et al.* Venomics of the Central American Lyre Snake Trimorphodon quadruplex

(Colubridae: Smith, 1941) from Costa Rica. *J. Proteomics* **220**, 103778 (2020).
3. De La Rosa, G. *et al.* Horse immunization with short-chain consensus α-neurotoxin generates antibodies against broad spectrum of elapid venomous species. *Nat. Commun.* **10**, 3642 (2019).
4. Modahl, C. M., Mukherjee, A. K. & Mackessy, S. P. An analysis of venom ontogeny and prey-specific toxicity in the Monocled Cobra (Naja kaouthia). *Toxicon* **119**, 8–20 (2016).
5. Naik, S. Intravenous snake bite: A catastrophic snake envenomation. *Ann. Afr. Med.* **22**, 239 (2023).
6. Kalek, I. S., *et al.* Synthetic development of a broadly neutralizing antibody against snake venom long-chain a-neurotoxins. *Sci. Trans. Med.* **16,** eadk1867 (2024).
7. Benard-Valle, M., *et al.* In vivo neutralization of coral snake venoms with an oligoclonal nanobody mixture in a murine challenge model. *Nat. Commun.* **15**, 4310 (2024).
8. Albulescu, L-O., *et al.* A therapeutic combination of two small molecule toxin inhibitors provides broad preclinical efficacy against viper snakebite. *Nat. Commun.* **11**, 6094 (2020).

• "We chose to target our design efforts against the neurotoxin edge β-strands (previously discovered monoclonal antibodies in contrast mimic the nAChR binding site (14,21,22), focusing on binding modes blocking neurotoxin binding to nAChRs through steric hindrance."

It would be helpful to the readers to know why this strategy was followed.

Response: *De novo* design of binders performs best when targeting rigid regions, rather than flexible loops. Thus, while the loops presented the functionally most relevant epitopes, we decided to focus on the proximal beta-sheets. By targeting the neurotoxin edge β-strands, we aimed for high-affinity and specific binding while blocking neurotoxin binding to nAChRs through steric hindrance. Top candidates were selected for *in vitro* validation based on their ability to interfere with toxin binding to nAChR. This rationale has been clarified in the second paragraph of the "Design of α-neurotoxin binding proteins" section in the revised text.

The modified text is shown below:

Design of α-neurotoxin binding proteins section

"We chose to target our design efforts against the neurotoxin edge β-strands, unlike previously discovered monoclonal antibodies that mimic the nAChR binding site[14,21,22]. To achieve functional neutralization, we focused on binding modes that block neurotoxin binding to nAChRs through steric hindrance. Secondary structure and block adjacency tensors were provided to the RFdiffusion model to specify desired β-strand interactions between the designed binder and target α-neurotoxins (see Methods). For each secondary structure tensor, interactions between one binder β-strand and a target neurotoxin β-strand were encoded in a block adjacency tensor, preconditioning RFdiffusion towards β-strand pairing complexes. Following backbone generation through RFdiffusion denoising trajectories, sequence design was carried out using ProteinMPNN, the resulting designs were filtered based on AF2 *initial guess*[40] and Rosetta metrics, and the most promising candidates selected for experimental characterization. Top candidates were selected for *in vitro* validation based on their ability to interfere with toxin binding to nAChR".

• Why wasn't a consensus sequence constructed for long chain α-cobratoxin? It would help the reader understand the rationale.

Response: The idea was to provide a proof of concept for different design approaches, i.e. 1) targeting a natural toxin for which a high resolution crystal structure exists (α-cobratoxin), 2) targeting a consensus toxin for which a high resolution crystal structure exists (ScNtx), and 3) targeting a novel consensus toxin for which we only had a AlphaFold 2 structure prediction (cytotoxin). To clarify these points for the reader, we have revised the second paragraph of the "MAIN" section and the fourth paragraph of the "Design of α-neurotoxin binding proteins" section accordingly.

The modified text is shown below:

MAIN section

"We explored the design of binders for both individual natural toxins and consensus toxins representing a family of toxin molecules, reasoning that binders to the latter could have broader neutralization activity".

Design of α-neurotoxin binding proteins section

"As a representative native long-chain α-neurotoxin…"

• The exact interaction modality between the protein inhibitor and long-chain 3FTx is mentioned. However, no such clarity is presented for short-chain 3FTx.

Response: We thank the reviewer for spotting this oversight - the text has been amended in the third paragraph of the "Design of α-neurotoxin binding proteins" section.

The modified text is shown below:

Design of α-neurotoxin binding proteins section

"The binder interacts with loop III of the neurotoxin, which is key for the toxin to bind to the muscle-type α7 nAChRs[42] (Fig. 3a). A β-strand in SHRT makes extensive backbone hydrogen bonds with an edge β-strand in the toxin (Fig. 3a, right inset), and tyrosine 45, which is located on an alpha helix in SHRT, forms a backbone hydrogen bond with cysteine 41 on ScNtx (Fig. 3a, left inset)".

• "fetal bovine serum at 37 °C and 5% and CO2." The second 'and' should be deleted.

Response: Agreed and addressed.

Referee #2 (Remarks to the Author):

This paper is about using recent protein design tools to design proteins that can neutralize snake venom toxins. Overall from a biology point of view the paper seems like a very solid contribution to a very important problem, although I should preface this that this area is outside my general area of expertise, so the rest of the review will focus on the design methodology itself and not discuss the experimental validation, as other reviewers will be better placed to judge this.

Response: The authors thank the reviewer for their time and constructive feedback.

The methodology uses RFDiffusion for designing the backbone, then uses ProteinMPNN to design the sequence and after filters designs using AlphaFold2 and Rosettafold.

• In fact this methodology is where one of my main criticisms of the paper comes in, the last sentence in the abstract is:

"Beyond snakebite, our computational design methodology should help democratize therapeutic discovery, particularly in resource-limited settings, by substantially reducing costs and resource requirements for development of therapies to neglected tropical diseases."
Which suggests that the paper claims that it is introducing a computational design methodology, however as far as I can tell this is just applying the methodology of the RFDiffusion publication to a new domain and as such I think there's no new methodology.

If there is a new methodology the authors should point out more carefully what they think is new about this methodology in the paper, otherwise the language in the abstract should be toned down to make clear that the paper doesn't propose a new methodology, parts of the main section are more clear that it's just using the method set out in RFDiffusion.

Response: The authors agree with the reviewer's comment and have amended the abstract to avoid confusion.

The modified text is shown below:

ABSTRACT section

"Beyond snakebite, our results highlight how computational design could help democratize therapeutic discovery, particularly in resource-limited settings, by substantially reducing costs and resource requirements for development of therapies to neglected tropical diseases".

• In general snake venom typically isn't a single protein, but rather a cocktail of a very large number of different toxic components. I think it would be useful if the authors could address why targeting a single protein is effective here.

Response: The reviewer is correct in that snake venoms typically constitute a complex mixture of multiple classes and isoforms of toxic proteins. Here, we demonstrate how some of the clinically most relevant toxins can be effectively neutralized. The development of fully designed antivenoms will require the identification of further binding components and presents the next goal which will be addressed in future studies. Recent studies have shown the efficacy of combining individual monoclonal antibodies (mAbs) to neutralize whole venoms[1,2]. We envision employing our *de novo* designed binders in a similar approach to achieve whole venom neutralization.

References:

1. Benard-Valle, M. *et al.* In vivo neutralization of coral snake venoms with an oligoclonal nanobody mixture in a murine challenge model. *Nat. Commun.* **15**, 4310 (2024).
2. Laustsen, A. H. *et al.* In vivo neutralization of dendrotoxin-mediated neurotoxicity of black mamba venom by oligoclonal human IgG antibodies. *Nat. Commun.* **9**, 3928 (2018).

• Also I wasn't entirely clear on the "In vivo protection" section, is this testing efficacy of the designed protein against the particular compound it was designed for or testing efficacy against what would be the use case, i.e. the snake venom which contains many other compounds. If it's the former I think this should be spelled out more clearly. Also how do the authors see this turning into a therapeutic? Would there be a need to design separate binders and manufacture them for each component in the venom?

Response: We agree with the reviewer, and the manuscript has been modified in the "In vivo protection" section for clarity. Current challenges in recombinant antivenom development, primarily focused on monoclonal antibodies, include time-consuming discovery and production of multiple antibodies, along with cost barriers in clinical trials and market entry[1]. Technologies facilitating rapid binder development, such as the one presented here, promise to significantly expedite this process. Furthermore, designed proteins offer cost-effective manufacturing via microbial fermentation and enhanced thermostability, reducing the need for costly cold-chain logistics. We argue that the next generation of antivenom therapies will focus not only on developing a mixture of *de novo* designed binders for specific toxins but also on *de novo* binders with broad recognition across a toxin class. Given the power of our technology to rapidly identify high-affinity binders against snake toxins and their therapeutic potential, we anticipate that *de novo* design could revolutionize snake envenomation treatment.

The modified text is shown below:

***In vivo* protection section**

“To evaluate the *in vivo* neutralizing efficacy of our neurotoxin-targeting protein binders, we monitored survival for 24 hours post-lethal neurotoxin challenge following administration of purified toxins”.

References:

1. Laustsen, A. H. Recombinant snake antivenoms get closer to the clinic. *Trends Immunol.* **45**, 225–227 (2024).

Aside from this the paper is very well written and easy to follow. The experimental validation of the designs looks very solid to me. However, as this paper is about applying RFDiffusion to designing binders of a particular component of snake venom, whether this paper is suitable for publication in Nature rests entirely on the application itself, as I’m not an expert on snake venoms I’d refer to other reviewers for this.

Referee #3 (Remarks to the Author):

The article by David Baker and co-workers elegantly presents the de novo design of proteins that neutralise snake venom 3FTXs. These toxins are very difficult to neutralise with animal-derived antibodies and are central to the pathophysiology of elapid snake envenomation. As this snake family claims tens of thousands of lives annually, the problem is timely and very relevant.
The study is very sound; the data is solid and robustly supports the conclusions. The work is groundbreaking - as far as I know, this is the first time de novo proteins have been designed to neutralise snake venom components. As such, this work opens up a whole new field of research in the agonising effort to help snakebite victims.
In summary, I have no doubt that this fantastic research is worthy of publication in Nature with minor changes. As a computational biochemist, I will not go into the more experimental aspects but will focus on the structural data and their fundamental significance.

Response: The authors would like to thank the reviewer for the positive and helpful feedback. We are equally excited about the opportunities *de novo* design holds in helping snakebite victims.

• Page 3: The authors state that " Synthetic genes encoding 44 designs targeting ScNTx were screened via yeast surface display (YSD), and one candidate was identified to bind to ScNTx with a dissociation constant (Kd) of 842 nM (…)". The reader would like to know the success rate of the method. Was this candidate protein the only submicromolar binder? Were there other protein candidates with similar affinities? The same question applies to affinity optimisation. What was the distribution of affinities among the 78 candidates of which one had an affinity of 0.9 nM?

Response: We thank the reviewer for the comment and have added more detail about the success rate of our method in the “Design of α-neurotoxin binding proteins” section. During initial binding screening using yeast surface display (YSD), only one binder was identified for both ScNTx (1/44) and α-cobratoxin (1/42). After partial diffusion optimization, several hits were identified. For ScNTx-binding proteins, 11 out of 78 candidates showed improved binding affinity compared to the initial hit based on YSD experiments. The top ScNTx-binding proteins were further characterized using bio-layer interferometry (BLI) and patch-clamp experiments. For α-cobratoxin-binding proteins, 6 out of 38 candidates demonstrated improved binding affinity compared to the initial hit, and the top α-cobratoxin-binding proteins were further characterized. BLI and functional neutralization data for the top binding candidates for ScNTx and α-cobratoxin are now included in Supplementary Figures S9 and S10.

The modified text is shown below:

Design of α-neurotoxin binding proteins section

"We targeted short-chain α-neurotoxins using a previously designed consensus toxin derived from elapid snakes (ScNtx)[41] as a representative template. Synthetic genes encoding 44 designs targeting ScNtx were screened via yeast surface display (YSD), and one candidate was identified to bind ScNtx with a dissociation constant (K_d) of 842 nM, as confirmed by bio-layer interferometry (BLI) (Supplementary Figure S1). Following partial diffusion optimization, 11 out of 78 designs had higher affinity than the initial hit (Supplementary Figure S9), with the best (SHRT) having a binding affinity of 0.9 nM, as determined by surface plasmon resonance (SPR)"

…

"As a representative native long-chain α-neurotoxin, we chose α-cobratoxin (P01391) from *Naja kaouthia*, one of the most extensively characterized toxins from the 3FTx family[43] (Figure 1). From 42 RFdiffusion designs against α-cobratoxin, one candidate had a binding affinity of 1.3 μM using BLI (Supplementary Figure S3). Partial diffusion optimization of the binding interface generated 38 designs, of which 6 had higher affinity than the initial hit (Supplementary Figure S10). The highest affinity binder, LNG, had a K_d of 1.9 nM as measured by SPR "**.**

• In the following paragraph, the authors describe the design of neutralising proteins against the monoclonal cobra 3FTxs. It is not immediately clear why they decided to do this after designing a neutralising protein for the consensus sequence toxin (although it becomes clear after seeing the results). A sentence stating the aims of this experiment would be useful here.

Response: The authors agree and have added an extra line at the end of the "MAIN" section to clarify the aim.

The modified text is shown below:

MAIN section

"We explored the design of binders for both individual natural toxins and consensus toxins representing a family of toxin molecules, reasoning that binders to the latter could have broader neutralization activity".

•Does the monocled cobra venom have multiple alpha-neurotoxin isoforms or a single isoform? A UniProt ID would help to unambiguously define the alpha-neurotoxin sequence.

Response: The authors apologize for this oversight - a UniProt ID has been added.

• Still on the same page, the authors state: "An arginine residue at position 33, located at the tip of loop II of α-cobratoxin, makes extensive electrostatic interactions with the binder". Do the authors have evidence that these interactions are important for affinity? Although Arg residues are often found in protein-protein binding hotspots, the Arg33 solvation free energy in the dissociated state may compensate for the binding free energy in the bound state. Therefore, establishing strong intermonomer interactions does not necessarily imply a positive contribution to affinity.

Response: We acknowledge the reviewer's comment regarding the role of Arg residues in protein binding interfaces. While we solved the crystal structure of our designed binder in complex with α-cobratoxin (Fig. 3b), we lack direct experimental evidence supporting the contribution of Arg33 to the binding affinity of our designed binder. Our reference to this interaction is based on previous studies that have extensively characterized the role of Arg33 in α-cobratoxin's interaction with the ligand-binding pocket of the acetylcholine binding protein (AChBP)[1]. These studies, along with mutagenesis analysis, have identified Arg33, located near the tip of loop II, as crucial in conferring toxin specificity. Given that our designed binder

primarily interacts with loop II of α-cobratoxin and Arg33 is positioned at the binding interface, we highlighted this interaction.

To address the reviewer's concern, we have revised the last paragraph of the "Design of α-neurotoxin binding proteins" section for greater clarity.

The modified text is shown below:

Design of α-neurotoxin binding proteins section

"Arginine 33, located at the tip of loop II of α-cobratoxin, forms extensive interactions with LNG (Fig. 3b, left inset); this toxin residue also makes extensive interactions with the AChBP".

References:

1. Bourne, Y., Talley, T. T., Hansen, S. B., Taylor, P. & Marchot, P. Crystal structure of a Cbtx–AChBP complex reveals essential interactions between snake α-neurotoxins and nicotinic receptors. *EMBO J.* **24**, 1512–1522 (2005).

• Page 4, first sentence - is the reference to Figure 1d correct?

Response: Thank you for highlighting this error - it should be 1b, and we have now corrected this.

• It would be interesting to retain the acetylcholine shown in Figures 1c and 1d, perhaps in a transparent material.

Response: Agreed and addressed.

• The mode of action of the antibodies is not immediately clear to me, especially after looking at Fig 1d. If the neutralizing proteins mimic the toxins, why are they not toxic themselves? How can acetylcholine bind and open the receptor in the presence of the bound neutralizing protein?

Response: We apologize for the confusion, the binders don't mimic the toxins - they precisely bind the regions of the toxins that bind and inhibit the receptor. This neutralizes the toxins and ensures the binders themselves don't interfere with acetylcholine or the receptor. We have now clarified the design rationale in the second paragraph of the "Design of α-neurotoxin binding proteins" section in the revised text.

The modified text is shown below:

Design of α-neurotoxin binding proteins section

"We chose to target our design efforts against the neurotoxin edge β-strands, unlike previously discovered monoclonal antibodies that mimic the nAChR binding site[14,21,22]. To achieve functional neutralization, we focused on binding modes that block neurotoxin binding to nAChRs through steric hindrance".

• Why do the CYTXs have a much lower affinity than previously designed neutralizing proteins?

Response: The lower affinity of CYTXs compared to previously designed neutralizing proteins can be attributed to differences in design strategies. Achieving ultra-high affinity binding to the flexible loop regions of cytotoxins is significantly more challenging than targeting rigid regions, such as the β-strands in α-neurotoxins. However, we successfully solved the crystal structure of CYTX in complex with *Naja pallida* cytotoxin (Fig. 3c), demonstrating that RFdiffusion can generate scaffolds shape-matched to protein targets. Despite the best CYTX binder exhibiting a binding affinity of 271 nM, this was sufficient to neutralize cytotoxicity in whole venoms, as shown by our functional *in vitro* experiments (Fig. 4c).

• Finally, it seems that obtaining interspecific neutralizing proteins by targeting the toxins rather than mimicking the receptors will be a challenge. What do the authors have to comment about this?

Response: Achieving broad neutralization capacity while retaining affinity is a challenge, but very much achievable as we observed for our cytotoxin binder (Fig. 4c). This has also been accomplished with monoclonal human antibodies[1]. Designing protein binders that neutralize toxins via receptor mimicry is certainly possible, as evidenced in Khalek et al[2]. Furthermore, generating receptor homologs through rational receptor modification has been previously achieved[3].

References:

1. Ledsgaard, L. *et al.* Discovery and optimization of a broadly-neutralizing human monoclonal antibody against long-chain α-neurotoxins from snakes. *Nat. Commun.* **14**, 682 (2023).
2. Khalek, I. S. *et al.* Synthetic development of a broadly neutralizing antibody against snake venom long-chain α-neurotoxins. *Sci. Transl. Med.* **16**, eadk1867 (2024).
3. Albulescu, L.-O. *et al.* A Decoy-Receptor Approach Using Nicotinic Acetylcholine Receptor Mimics Reveals Their Potential as Novel Therapeutics Against Neurotoxic Snakebite. *Front. Pharmacol.* **10**, 848 (2019).

Referees' comments:

Referee #1 (Remarks to the Author):

The authors have sufficiently addressed all my comments, and I am happy with the revised version of the manuscript. I recommend the publication of this very exciting, well-planned, and well-executed study in Nature.

Response: The authors would like to thank the reviewer for taking the time to go through this manuscript. We are pleased that our work is being considered for publication in *Nature* and appreciate your recommendation.

Referee #2 (Remarks to the Author):

I want to thank the authors for the detailed feedback. The authors addressed all my questions satisfactorily.

Response: We thank the reviewer for their thoughtful comments and for taking the time to review our manuscript. We are pleased that our responses have satisfactorily addressed all of the reviewer's questions.

Referee #3 (Remarks to the Author):

The authors have addressed all my questions and comments except one, which still puzzles me.

Still on the mechanism of action of the neutralizing proteins - if they block acetylcholine binding, as shown in Figure 1c, why don't they inhibit nicotinic acetylcholine receptors and display the same neurotoxic effect as the snake toxins?

Apart from that, this excellent scientific paper is ready for publication. It fully meets Nature's strict criteria for quality and impact.

Response: We apologize for the confusion. The binders do not block acetylcholine binding; instead, they bind to the neurotoxins, thereby preventing neurotoxin binding to nAChRs through steric hindrance. To clarify, Fig. 1c illustrates how neurotoxins alone inhibit nAChRs. We have revised the text, specifically in the second paragraph of the 'Design of α-neurotoxin binding proteins' section, to clarify the design rationale.

The modified text is shown below:

Design of α-neurotoxin binding proteins section

“**..**Top candidates were selected for *in vitro* validation based on their ability to interfere with toxin binding to nAChR, guided by structural alignments comparing the neurotoxin-nAChR complex with and without the designed binder”
